# Long-term comparative trajectories of health, behavioral, and economic outcomes: From pre-diagnosis to survivorship

**DOI:** 10.1371/journal.pone.0357917

**Published:** 2026-09-09

**Authors:** Hui G. Cheng, Livingstone Aduse-Poku, Oxana Palesh, Susan Hong

**Affiliations:** 1 Massey Comprehensive Cancer Center, Virginia Commonwealth University, Richmond, Virginia, United States of America; 2 Department of Psychiatry, Virginia Commonwealth University School of Medicine, Richmond, Virginia, United States of America; 3 Division of Hematology, Oncology and Palliative Care, Department of Internal Medicine, Virginia Commonwealth University School of Medicine, Richmond, Virginia, United States of America; Touro University California College of Pharmacy, UNITED STATES OF AMERICA

## Abstract

**Purpose:**

Although cancer survivorship involves interconnected changes in health, health behaviors, and economic well-being, few studies have comprehensively examined how these outcomes evolve before and after a cancer diagnosis within the same population over time. Using population-based longitudinal data, we characterized trajectories before and after incident cancer diagnosis and compared them with those of matched individuals without cancer.

**Methods:**

Using 15 waves (1992–2022) of the Health and Retirement Study, we identified 5,423 incident cancer cases and matched them to up to four controls (n = 17,530) using exact matching on sex and race/ethnicity and propensity score matching on age and entry wave (caliper = 0.1). Outcomes included health (self-rated health, functional limitations, depressive symptoms, cognition), lifestyle (BMI, smoking, drinking, physical activity), and economic measures (employment, out-of-pocket medical spending). Using a time-centered design, all measures were aligned to the wave of incident cancer diagnosis (t₀), and outcome trajectories were estimated from up to 24 years before to 24 years after diagnosis. Differences in post-diagnosis changes between cancer cases and matched controls were assessed using fixed-effects difference-in-differences models.

**Results:**

Cancer diagnosis was associated with immediate declines in self-rated health (−19%) and increased functional limitations (+44%) at t₀, followed by partial recovery but persistent deficits. Depressive symptoms spiked and remained elevated for four years. Smoking declined at diagnosis and remained lower, while changes in BMI and alcohol use were more transient. Physical activity declined and remained suppressed for six years. Employment dropped sharply, and out-of-pocket spending rose by $2,382 (95% CI: $1,705 to $3,061), with disparities persisting for six years. Mortality risk remained elevated and widened through year six. Notably, cancer survivors exhibited higher post-diagnosis cognitive functioning.

**Conclusion:**

Cancer diagnosis is associated with profound, multidimensional disruptions, many of which persist for years. These findings underscore the need for comprehensive survivorship care, including mental health services, rehabilitation, lifestyle counseling, and financial support, to address the long-term burden.

## Introduction

Cancer survivorship has become a major public health priority as the number of individuals living with a history of cancer continues to rise [1]. As of January 1, 2025, an estimated 18.6 million cancer survivors reside in the United States, representing approximately 5.4% of the population, and this number is projected to exceed 22 million by 2035 [2]. This growth reflects both population aging and advances in early detection and treatment that have improved survival [1,2]. However, survivorship is often accompanied by acute and lasting effects of cancer and its treatment, including physical and cognitive impairments [3–5], functional limitations [6,7], psychological distress [5,8–10], lifestyle changes [9,11], and employment and financial hardship [12,13]. These challenges can persist for years and significantly affect quality of life and mortality risk.

Despite the growing population of cancer survivors, gaps remain in understanding the long-term trajectories of health, behavioral, and economic outcomes. Most prior studies have focused on short-term outcomes following diagnosis or examined single domains such as physical functioning or mental health. Few have comprehensively assessed how these outcomes evolve over extended periods, particularly in comparison to individuals without cancer. Such evidence is critical for informing multidisciplinary and time-sensitive survivorship care planning across the survivorship continuum emphasized by leading organizations such as the American Society of Clinical Oncology (ASCO) and the National Cancer Institute (NCI) [14].

To address these gaps, we conducted a longitudinal matched cohort study using data from the Health and Retirement Study (HRS), a nationally representative sample of U.S. adults aged 50 and older. By constructing trajectories spanning up to two decades before and after cancer diagnosis, we aimed to

quantitatively characterize longitudinal patterns in health, behavioral, and economic outcomes among individuals with incident cancer before and after diagnosis and compare them with those of matched individuals without cancer in order to inform survivorship care strategies and policy efforts.

The current study directly addresses several key gaps in cancer survivorship research [15]. First, by including a matched cancer-free comparison group, we are able to gain a clearer understanding of survivorship outcomes within the context of normal aging and lifestyle patterns [16]. Second, the use of repeated measures spanning up to two decades before and after diagnosis allows for the construction of long-term trajectories, shedding light on long-term trajectories to complement finer-grained, shorter-term clinical studies. Third, the inclusion of validated assessments across multiple domains of common outcomes enhances the rigor and relevance of the findings, contributing to efforts to standardize survivorship research and inform targeted interventions. Fourth, the use of a large, nationally representative sample improves generalizability to U.S. adults aged 50 and older. Finally, by focusing on incident cancer cases, the study minimizes survival bias associated with prior cancer diagnoses.

## Materials and methods

### Study design, study population, and data source

We used a matched cohort study design to assess longitudinal patterns associated with cancer diagnosis by examining trajectories of relevant variables up to 24 years before and after diagnosis. This analysis used data from 15 waves of the Health and Retirement Study (HRS) [17], a nationally representative longitudinal study of Americans aged 50 and older, conducted biennially since 1992.

The inception HRS cohort (1992) comprised of individuals born between 1931 and 1941, drawn using a multistage probability sampling approach. The 1993 Asset and Health Dynamics Among the Oldest Old (AHEAD) study of a cohort of individuals aged 70 and older (born 1890–1923) were merged with the original HRS cohort in 1998. In order to ensure the representativeness of the sample for the USA population over age 50, two new cohorts were enrolled: those who were born between 1924 and 1930 (the Children of the Depression), and those born between 1942 and 1947 (the War Babies). Additional birth cohorts were then added every six years to replenish the sample and to maintain representativeness of the aging U.S. population [18].

In this study, we identified incident cancer cases, defined as participants who had never had cancer at baseline assessment and developed cancer during the follow-up. Controls were participants who had never had cancer up to the last follow-up in 2022 and were matched with incident cancer cases based on sex, age, race/ethnicity, and HRS entry wave. We included only control participants who were alive at the time their matched cancer case was diagnosed. This design enabled us to examine longitudinal trajectories of key variables before and after cancer diagnosis.

### Assessment and outcomes

The HRS employed a mixed-mode data collection approach. Baseline interviews were typically conducted face-to-face. Follow-up interviews were primarily conducted using phone interviews prior to 2004. Since 2006, half of the sample completed follow-up interviews in-person, which included expanded modules; the other half sample completed core modules only via phone interviews. The half-samples alternated waves, so expanded content was available for half of the sample at each wave and every other wave longitudinally for each participant.

Vital status was tracked through interviews as well as linkages to the National Death index.

In this study, we focused on variables relevant to cancer survivorship that were most consistently assessed. These variables can be grouped into 3 domains: health-related (self-rated health, functional limitations [19,20], depressive symptoms [21], and cognitive functioning), lifestyles (body mass index, smoking status, drinking status, and physical activities), work and expenditure (work status and out-of-pocket medical spending). The reference period was the previous 2 years for out-of-pocket medical spending, the previous 3 months for drinking status, and the previous week for depressive symptoms; all other variables reflected participants’ current status at the time of interview. Table 1 presents details of these variables and their validated assessments [19–23].

**Table 1:** Outcome Variables.

Domains	Variable	Assessment	Responses
Health-related	Death	Follow-up interview and linkage to National Death Index	Yes or No
	Self-rated health	“Would you say your health is excellent, very good, good, fair, or poor?”	Poor Fair Good Very good Excellent
	Functional limitation – activities of daily living	Katz Index of Independence in Activities of Daily Living [19] “Because of a health or memory problem, do you have any difficulty with…?” A question each for bathing, dressing, eating, getting in or out of bed, walking across a room, and using the toilet.	Any “yes” responses to each question (Yes) or all “no” responses (No)
	Functional limitation – instrumental activities of daily living	Lawton Instrumental Activities of Daily Living [20] “Because of a health or memory problem, do you have any difficulty with…?” A question each for using the telephone, managing money, taking medications, shopping for groceries, and preparing meals.	Any “yes” responses to each question (Yes) or all “no” responses (No)
	Depressive symptoms	8 items from the Center for Epidemiologic Studies Depression Scale [21] “During the past week, did you …?” A question each for feeling depressed, everything was an effort, sleep was restless, lonely, sad, could not get going, enjoying life, and happy?	Number of “yes” responses to each question, ranging from 0 to 8 (enjoying life and feeling happy were reversely coded)
	Cognitive functioning	Composite score based on: immediate word recall (10 words), delayed word recall (10 words), serial 7s subtraction (up to 5 points), counting backwards from 20, naming today’s date, naming the President and Vice President, and vocabulary questions (e.g., naming objects)	Sum of all items Range: 0 to 35
Lifestyle	Body mass index	Self-reported weight divided by the square of height Survey questions: “How tall are you without shoes?” “How much do you weigh without clothes or shoes?	Numeric
	Current smoking	“Do you smoke cigarettes now?”	Yes or No
	Current drinking	“In the last 3 months, how often did you drink any alcoholic beverages?”	Yes (any) or No (none)
	Physical activities	“How often do you take part in…?” A question each for vigorous physical activity (e.g., running, swimming, cycling), moderate activity (e.g., gardening, walking at a moderate pace), or light activity (e.g., stretching, slow walking).	Hardly ever or never 1-3 times a month Once a week More than once a week Every day
Work and finance	Work status	“Are you currently working for pay?”	Yes or No
	Out-of-pocket medical expenditure	“About how much did you pay out-of-pocket for medical care in the past 2 years, including doctor visits, hospital stays, prescriptions, and other health services?”	Numeric in $

### Statistical analysis

#### Descriptive analysis.

In this study, we used a time-centered approach to align measures by the study wave when the incident cancer was reported, denoted as *t*_*0*_. Measurements before cancer diagnosis were denoted as *t-1*, *t-2*, etc., and those after cancer diagnosis were denoted as *t + 1*, *t + 2*, etc. Participants with incident cancer were then matched with controls using exact match on sex (male or female) and race/ethnicity (Hispanic, non-Hispanic White, non-Hispanic Black, and other) and propensity score matching on age (in years) and HRS entry wave with a caliper of 0.1. Each case was matched with up to 4 controls. Controls who deceased by *t*_*0*_ were excluded. Because not all cases had 4 matched controls, weights were used in analysis to account for uneven number of controls for each case. Taylor Series Linearization was used for variance estimation to account for potential clustering as a result of the multi-stage sampling.

We first described baseline characteristics and assessed the balance between the two groups. To produce trajectories of variables of interest, we calculated the mean (for numeric variables such as body mass index) or proportion (for binary variables such as current smoking) of the aforementioned outcomes and their 95% confidence intervals for the incident cancer and control groups at each time points. Because *t*_*0*_ can be any study wave from wave 2–15, the theoretical maximum retrospective and prospective window is 28 years; however, because the number of participants decrease as *t* moves further away from *t*_*0*_, we truncated the window at 24 years when there were at least 100 participants in the incident cancer group. Frequencies of physical activities were first assessed in wave 7, and out-of-pocket medical expenditure was first assessed in wave 10, so the retrospective and prospective windows were shorter for these variables. Paired t-tests were used to evaluate differences between cancer cases and matched controls. Because these comparisons were descriptive, we did not adjust for type I error.

For these trajectory estimates, we conducted two sets of sensitivity analyses. First, we used 1:1 exact matching on all variables. Second, we incorporated matching weights with HRS weights that accounted for selection probability and attrition.

#### Difference-in-Differences analysis.

To formally evaluate changes in health, behavioral, and economic outcomes associated with cancer diagnosis, we estimated difference-in-differences (DiD) models using a two-way fixed-effects framework. For each outcome, we estimated the following model:


$$\begin{array}{c} Y_{it}=\beta_{\text{0}}+\sum_{k}\beta_{k}\left({Cancer}_{i}\times {Period}_{kt}\right)+\alpha_{i}+\gamma_{t}+\varepsilon_{it} \end{array}$$

where *Y*_*it*_ represents the outcome for individua *i* at survey wave *t*; *Cancer*_*i*_ indicates group (case or control); *Period*_*kt*_ denotes time periods relative to diagnosis (pre-diagnosis, diagnosis wave, 1–5 years post-diagnosis, and >5 years post-diagnosis); *α*_*i*_ represents individual fixed effects; and *γ*_*t*_ represents time-period fixed effects. Individual fixed effects account for all observed and unobserved time-invariant participant characteristics, while time fixed effects account for secular changes common to both groups.

The interaction terms between cancer status and each post-diagnosis period estimated the differential change in outcomes among cancer survivors relative to matched controls compared with the pre-diagnosis period. Standard errors were two-way clustered at the matched-pair and individual levels to account for both the matched study design and within-person correlation arising from repeated observations across waves. For the 15 outcomes examined, p-values were adjusted for multiple testing using the Benjamini-Hochberg false discovery rate procedure [24]. To assess the parallel trends assumption, we estimated wave-specific interactions between cancer status and time during the pre-diagnosis period and performing a joint Wald test of these coefficients.

All analyses were conducted using R. This study utilized publicly available, de-identified data from the HRS. The HRS received ethical approval from the University of Michigan Health Sciences and Behavioral Sciences Institutional Review Board.

## Results

A total of 5431 incident cancer cases occurred during the 30 years of study period, of whom 7 had missing value on race/ethnicity. Of the remaining 5424 cases, 5423 were successfully matched to 17530 controls. One case could not be matched according to the prespecified matching criteria and was excluded from subsequent analyses. The case group consisted of 2771 (51%) males and 2652 (49%) females; 427 Hispanic (8%), 4067 (75%) non-Hispanic white, 820 non-Hispanic Black (15%), and 109 (2%) non-Hispanic other race/ethnicity individuals; median age was 57 years at baseline assessment and 70 years at the wave of cancer incidence. The two groups were well balanced on these characteristics (*p > 0.999*), indicating successful matching. The two groups were also balanced with respect to marital status (60.3% and 61.7% were married in case and control groups, *p = 0.150*), rural/urban residence (49.0% and 48.2% urban residence in case and control groups, *p = 0.290*), and level of education (43% and 42% with some college or above education, *p = 0.170*).

**Fig 1:**
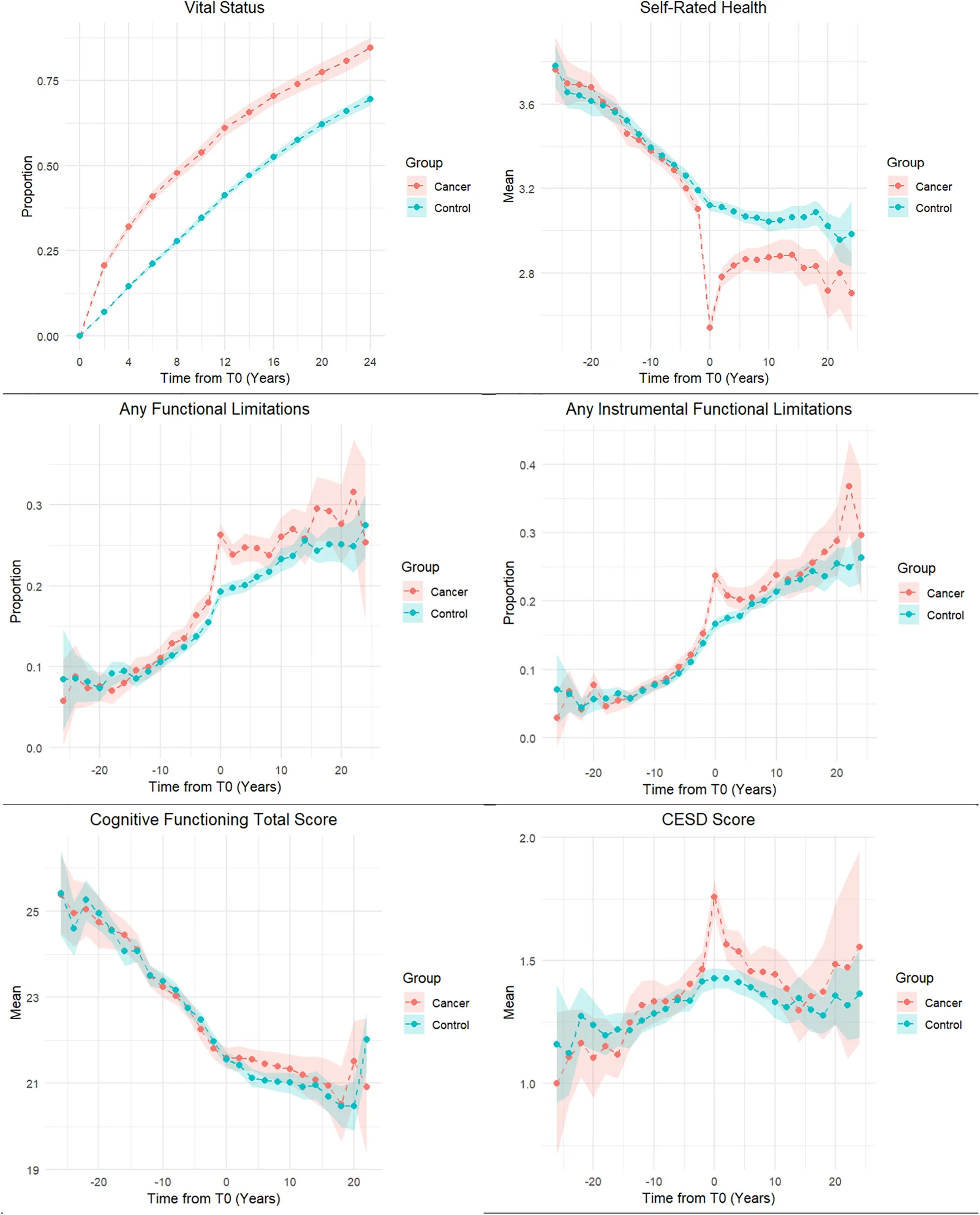
Trajectories of vital status and health-related outcomes before and after cancer diagnosis among individuals diagnosed with incident cancer and their matched controls. Shaded area represents 95% confidence intervals. The y-axis does not start at zero to facilitate visualization of group differences; therefore, visual differences may appear larger than the corresponding absolute differences.

Individuals diagnosed with incident cancer were more likely to experience mortality compared to their matched controls (Fig 1, Supporting Information S1 Table). The mortality gaps continued to widen from 14% (21% in the cancer group and 7% in the control group) in year 2–20% (61% in the cancer group and 41% in the control group) in year 6 post-diagnosis and then plateaued.

In addition to excess in mortality, cancer survivors also experienced acute and persistent health declines post-diagnosis. At the time of cancer diagnosis, self-rated health showed a drastic decrease (from an average of 3.1 [good] at *t-1* to 2.5 [fair-to-good] at *t*_*0*_), which returned to 2.8 two years post-diagnosis. Nonetheless, cancer survivors experienced persistently poorer health compared to their matched controls during the entire follow-up [Fig 1]. Of note, the two groups had almost identical pre-diagnosis ratings and trends until 2 years pre-diagnosis (i.e., *t-1)* when the cancer group had slightly poorer health compared to controls (3.1 vs. 3.2; *p < 0.001* from paired t-test). Consistent with the descriptive trajectories, DiD analyses showed a marked decline in self-rated health at diagnosis (β = −0.54; 95% CI, −0.57 to −0.51), followed by partial recovery. Nevertheless, self-rated health remained significantly below pre-diagnosis levels during both 1–5 years post-diagnosis (β = −0.31; 95% CI, −0.35 to −0.28) and more than 5 years post-diagnosis (β = −0.21; 95% CI, −0.25 to −0.18; Table 2). Similar trends were observed for functional limitations. The proportion of participants experiencing functional limitations increased from 18% at *t-1*–26% at *t*_*0*_ (an 8-percentage-point [pp] increase; 44% relative increase) for basic activities and from 15% to 24% (a 9-pp increase; 60% relative increase) for instrumental activities. Depressive symptoms (score ranging from 0 to 8) increased at the time of cancer diagnosis (from 1.46 at *t-1* to 1.76 at *t*_*0*_*,* a 20% increase in the cancer group) and remained elevated until at least 4 years post-diagnosis. Consistent with these descriptive findings, DiD analyses indicated significant worsening in functional limitations and depressive symptoms at the wave of diagnosis, followed by modest recovery; however, these outcomes remained significantly worse than pre-diagnosis levels throughout the post-diagnosis period (Table 2). With respect to cognitive functioning, the DiD analysis revealed a significant decline in cognitive functioning at the wave of diagnosis (β = −0.30; 95% CI= −0.47, −0.13). However, this deficit was no longer evident during the 1–5 years following diagnosis, and cognitive functioning became significantly higher among cancer survivors than controls more than 5 years after diagnosis (β = 0.36; 95% CI = 0.15, 0.57). To assess the potential role of survival, we used a mixed-effects model to estimate group differences in the association between cognitive function at the prior wave and death at the current wave. The results showed a stronger inverse association among controls, with ORs of 0.89 (95% CI = 0.88, 0.89) and 0.92 (95% CI = 0.91, 0.94) in the control and cancer groups, respectively (*p < 0.001* for the interaction term).

**Table 2:** Pre-Diagnosis Differences and Estimates from Difference-in-Difference Model for t_0_ and Post-Diagnosis Compared to Pre-Diagnosis.

Outcome	Pre-diagnosis differences (reference)*	Estimate for t0 [95% CI]	*p_t0_*	Estimate for Post Period ≤ 5 Years [95% CI]	*p_post≤5 yr_*	Estimate for Post Period > 5 Years [95% CI]	*p_post>5 yr_*
Self-Rated Health	-0.05	-0.54 [-0.57, -0.51]	<0.001	-0.31 [-0.35, -0.28]	<0.001	-0.21 [-0.25, -0.18]	<0.001
Functional Limitation	2%	6% [5%, 8%]	<0.001	5% [3%, 6%]	<0.001	3% [2%, 5%]	<0.001
Instrumental Functional Limitation	1%	7% [6%, 8%]	<0.001	4% [3%, 5%]	<0.001	2% [1%, 4%]	0.001
CESD score	0.06	0.32 [0.27, 0.38]	<0.001	0.17 [0.11, 0.22]	<0.001	0.18 [0.11, 0.24]	<0.001
Cognitive Functioning	0.04	-0.30 [-0.47, -0.13]	0.001	-0.03 [-0.21, 0.15]	0.762	0.36 [0.15, 0.57]	0.002
BMI	0.30	-0.57 [-0.67, -0.47]	<0.001	-0.15 [-0.26, -0.03]	0.014	-0.16 [-0.32, -0.01]	0.047
Obesity	2%	-3% [-4%, -2%]	<0.001	0% [-1%, 1%]	0.762	-1% [-3%, <1%]	0.094
Current Smoking	3%	-5% [-6%, -4%]	<0.001	-4% [-4%, -3%]	<0.001	-1% [-2%, >-1%]	0.047
Current Drinking	1%	-5% [-6%, -4%]	<0.001	-3% [-4%, -2%]	<0.001	-3% [-4%, -1%]	0.001
# of Drinking Days	0.07	-0.20 [-0.25, -0.15]	<0.001	-0.13 [-0.18, -0.08]	<0.001	-0.11 [-0.18, -0.05]	0.001
Light Activity	-0.03	-0.19 [-0.24, -0.14]	<0.001	-0.11 [-0.16, -0.06]	<0.001	-0.09 [-0.15, -0.03]	0.003
Moderate Activity	-0.09	-0.19 [-0.24, -0.13]	<0.001	-0.19 [-0.24, -0.13]	<0.001	-0.16 [-0.22, -0.09]	<0.001
Vigorous Activity	-0.04	-0.17 [-0.22, -0.12]	<0.001	-0.15 [-0.20, -0.10]	<0.001	-0.09 [-0.16, -0.03]	0.007
Work status	-2%	-7% [-8%, -6%]	<0.001	-6% [-7%, -4%]	<0.001	-3% [-5%, -1%]	0.001
Out-of-Pocket Medical Spending ($)	223	2118 [1435, 2801]	<0.001	646 [122, 1170]	0.018	612 [-67, 1291]	0.083

CESD: Center for Epidemiologic Studies Depression Scale.

* Tests of pre-diagnosis trends showed no significant differences in outcome trajectories between cancer survivors and matched controls before diagnosis (joint test p>0.05 for all outcomes except for self-rated health (p=0.017), instrumental functional limitation (p=0.003), and light activity (p=0.003).

**Fig 2:**
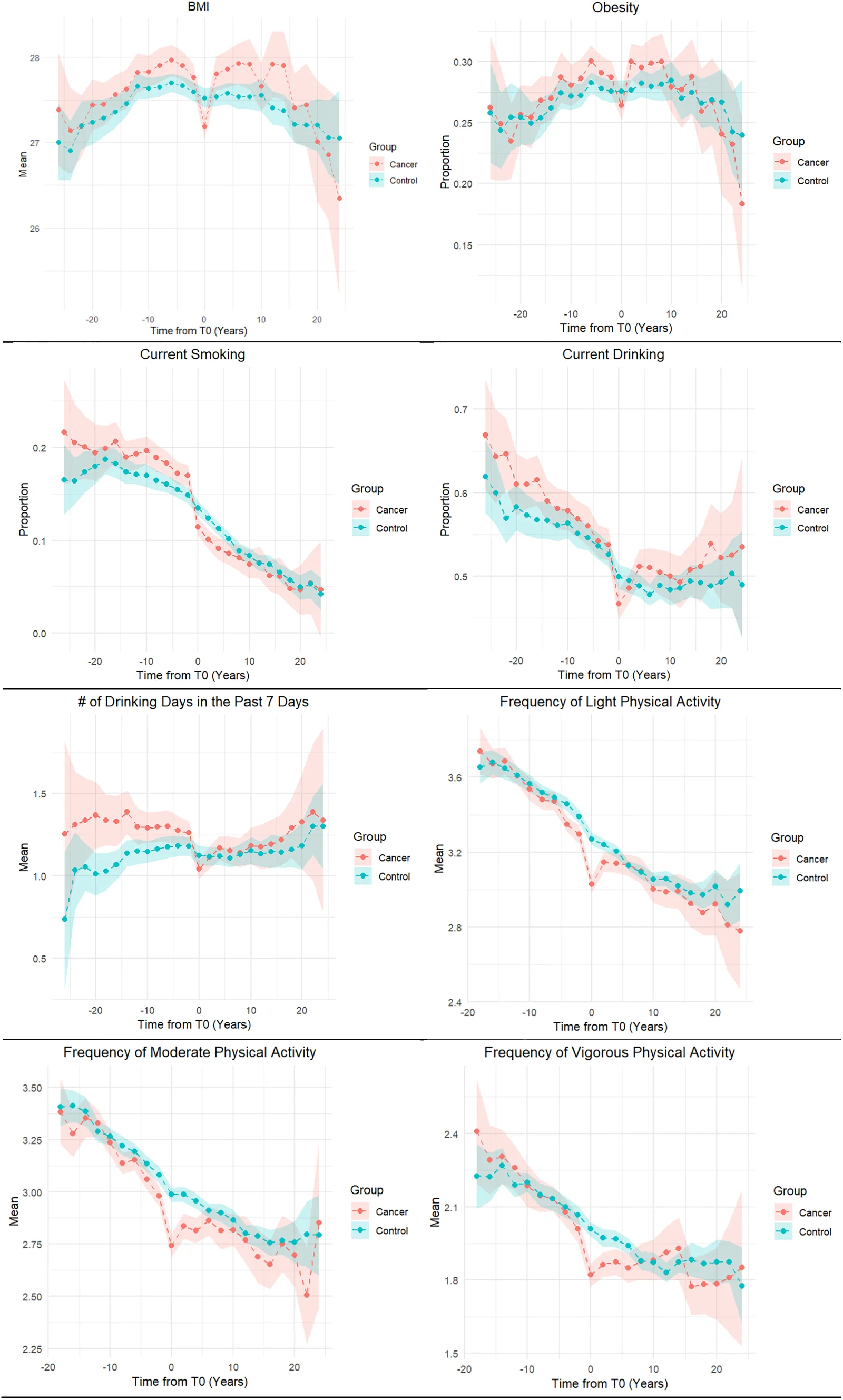
Trajectories of Body Mass Index and health behaviors before and after cancer diagnosis among individuals diagnosed with incident cancer and their matched controls. Shaded area represents 95% confidence intervals. The y-axis does not start at zero to facilitate visualization of group differences; therefore, visual differences may appear larger than the corresponding absolute differences.

With respect to lifestyle related factors, individuals with incident cancer tended to have higher BMI and be more likely to smoke cigarettes and drink alcohol pre-diagnosis (Fig 2 and Table 2). The two groups had similar frequencies of physical activities up to 4 years pre-diagnosis, followed by lower frequencies of physical activities in the cancer group. BMI decreased (from 27.8 to 27.2) at the time of cancer diagnosis and returned to the pre-diagnosis level at *t1*, and obesity showed a similar pattern. The prevalence of smoking also dropped at the time of cancer diagnosis and it remained lower compared to the control group post-diagnosis until both groups had very low prevalence approximately 10 years after *t*_*0*_. The prevalence of drinking also decreased at the time of cancer diagnosis and then returned to almost comparable levels as controls 2 years post-diagnosis. Frequencies of physical activities dropped at the time of cancer diagnosis and did not return to comparable levels as the controls until 6 years post-diagnosis. Results from DiD models were generally congruent with these observations.

**Fig 3:**
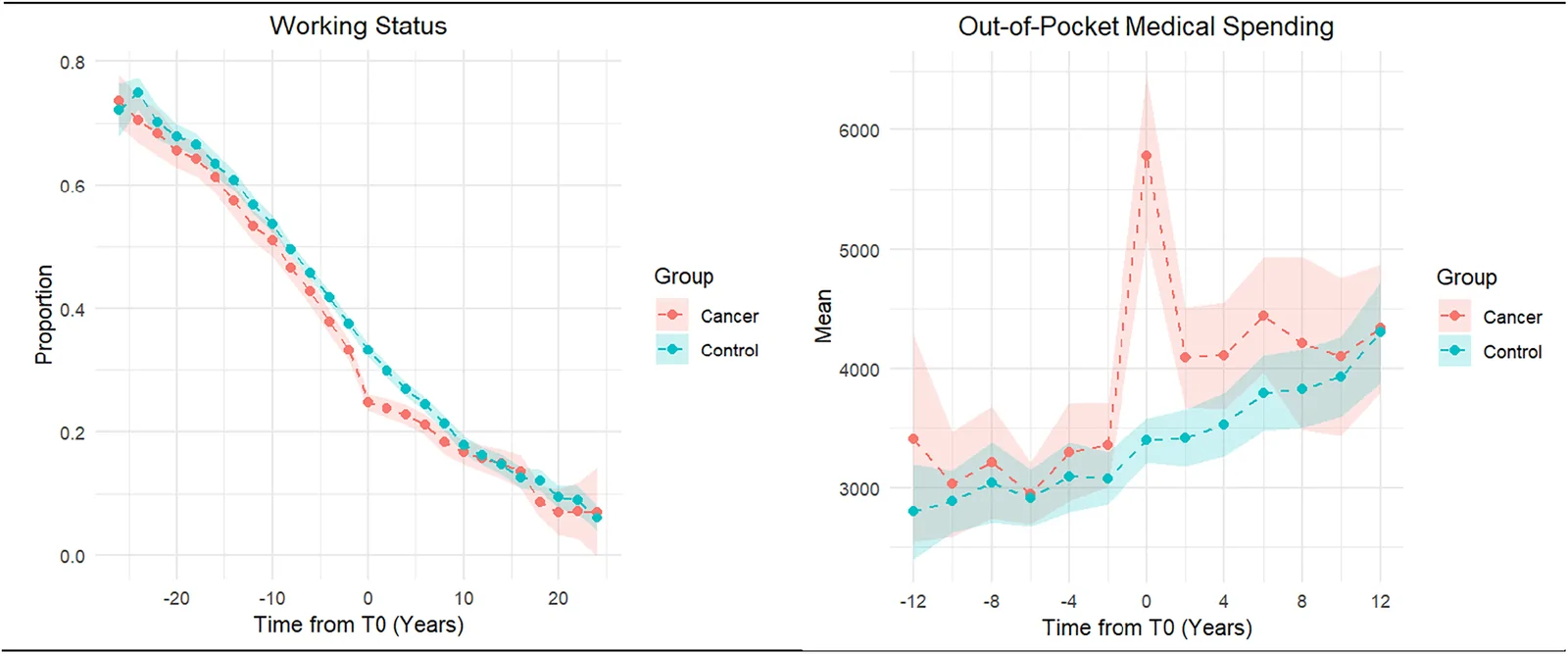
Trajectories of work status and out-pocket medical spending before and after cancer diagnosis among individuals diagnosed with incident cancer and their matched controls. Shaded area represents 95% confidence intervals. The y-axis does not start at zero to facilitate visualization of group differences; therefore, visual differences may appear larger than the corresponding absolute differences.

The proportion of working for pay is slightly lower in the incident cancer group pre-diagnosis (Fig 3 and Table 2). The difference grew substantially at the wave of cancer diagnosis, followed by a slow closing until 8 years post-diagnosis when the difference returned to pre-diagnosis levels. The DiD results were consistent with this observed pattern showing a substantial decline at the wave of diagnosis (−7%; 95% CI = −8%, −6%), followed by partial recovery that remained significantly below pre-diagnosis levels during the post-diagnosis period (Table 2). With respect to past-2-year out-of-pocket medical spending, a large spike occurred at the wave when cancer diagnosis occurred among the cancer group mounting to a $2382 difference (95% CI: $1,705 to $3,061; *p < 0.001* from paired t-test) between the two groups despite similar levels pre-diagnosis. The gap did not completely close until 8 years after the cancer diagnosis. Consistent with these descriptive patterns, the DiD analysis identified a large increase in out-of-pocket medical spending at the wave of diagnosis, followed by a smaller but still elevated level of spending during the 1–5 years post-diagnosis period (Table 2). Sensitivity analyses produced similar patterns (Supporting Information S1 and S2 Figs).

## Discussion

In this longitudinal matched cohort study spanning over two decades before and after cancer diagnosis, we provide a comprehensive view of the multifaceted and enduring changes associated with cancer diagnosis on survivors’ lives beyond clinical outcomes. By comparing individuals with incident cancer to matched controls, we reconstructed the natural history surrounding a cancer diagnosis and quantified changes associated with cancer diagnosis across health, lifestyle, and economic domains. These findings align with the ASCO’s and NCI’s survivorship care planning principles, which emphasize a multidisciplinary, dynamic, and time-sensitive approach to survivorship care [14,25].

### Findings and Implications

The most pronounced changes occurred within the 2 years of diagnosis, affecting all domains studied. The large increase in the loss of functioning necessary for independent living highlight the burden associated with cancer diagnosis as well as people around them. Self-management training can help preserve independence [26] and should be incorporated into survivorship care immediately upon diagnosis along with resources available for caregivers [27]. The marked spike in depressive symptoms at the wave of diagnosis highlights the need for incorporating mental health care during the active treatment phase given that depression can reduce the adherence to cancer treatment as well as the effectiveness of treatment [28], and there is drastically heightened suicide risk within 3 months of diagnosis [29].

Post treatment, there is persistent health declines for up to 30 years post-diagnosis, substantial functional losses lasting up to a decade, and elevated depressive symptoms, employment disruptions, and out-of-pocket medical expenses for up to four years. These findings extend the existing literature on the long-term outcomes of a cancer diagnosis by providing a time-sensitive picture [6,30–33].

In the meantime, reduction in BMI and drinking around cancer diagnosis were relatively transient. The transient decline in BMI observed around the time of diagnosis may reflect cancer-related weight loss or treatment effects rather than intentional behavioral changes. Notably, the cancer group had higher prevalence of obesity and alcohol drinking before cancer diagnosis, which is consistent with the large body of literature showing that excessive weight and alcohol drinking are causes of many malignancies [10,34]. Particularly, despite the widely held belief that moderate drinking is beneficial for health, recent evidence has shown a monotonic relationship between alcohol consumption and cancer risk [35–38]. Given the weight of the evidence, the US Surgeon General’s Advisory recommended including cancer in the warning label on alcoholic beverages, counseling patients about alcohol as a cancer risk factor, and promoting alcohol intervention strategies in clinical settings [39]. And many health organizations, including the World Health Organization [40] and the American Cancer Society [41], clarified there is no safe level of drinking with respect to cancer. In this context, the > 50% drinking prevalence and ~30% obesity among cancer survivors highlight the urgent need for lifestyle interventions in survivorship care.

Physical activity is beneficial to physical and mental health as well as overall quality of life among cancer survivors [42]. It also plays a critical role for rebuilding muscle mass after cancer treatment [43,44]. Recognizing the role of physical activities, the American Cancer Society recommend at least 150 minutes/week of moderate-intensity activity for cancer survivors [41]. In this study, we observed that the reduced level of physical activities upon diagnosis did not return to a similar level as controls until 6 years after cancer diagnosis. More importantly, the average frequency of moderate activities is less than weekly, which is likely well below the recommendation.

Smoking is linked to many negative health outcomes, including cancer [45]. In this study, the sustained higher quitting rates among cancer survivors reflects a great accomplishment of concerted smoking cessation efforts. Nonetheless, 10% of cancer survivors were still smoking after cancer diagnosis, which signifies continuing efforts for smoking cessation.

These intercorrelated factors may help explain the observed higher mortality among cancer survivors, with gaps widening over time and plateauing approximately 6 years post-diagnosis, likely driven by both cancer-related and noncancer causes including cardiovascular diseases, diabetes, and suicide [46–51].

Taken together, these findings highlight large gaps in cancer survivorship care and opportunities to improve the quality-of-life post treatment. At diagnosis, care plans should incorporate mental health screening and strategies to preserve daily functioning. Post-treatment, it is necessary to continue to screen for functional limitations and mental wellbeing, with timely referrals to rehabilitation and mental health services. Lifestyle counseling (including smoking cessation, physical activity, and weight management) is critical during the period following active treatment given lifestyle factors account for 44% of cancer deaths [52], and adherence to a healthy lifestyle is associated with substantial lower risks of cancer recurrence [53–56]. Post-treatment cancer care should also include financial navigation support to reduce out-of-pocket costs and vocational rehabilitation services to facilitate workforce reintegration [57,58]. At the macro-level, policy-level interventions to reduce out-of-pocket costs and support employment continuity are necessary to mitigate financial burdens during survivorship. Here, we echo the call for a comprehensive, multi-faceted cancer survivorship care framework [14]. Further, many aspects of survivorship care can be delivered virtually. Proper use of fast evolving technologies can help ensure equitable access to tailored survivorship care and provide real-time fine-grained empirical data for the optimization of care strategies. Local community-based centers can be an effective way to deliver integrated care.

### Cancer diagnosis and cognitive functioning

One unexpected finding is the tendency of better post-diagnosis cognitive functioning among cancer survivors compared with controls. Although chemotherapy-related cognitive impairment is well documented, evidence from long-term studies is sparse, and literature reviews have identified methodological limitations such as the lack of control groups and baseline assessments [4,59]. Our study filled these gaps in knowledge. One possible explanation is selective survival, whereby cancer survivors with poorer cognitive functioning are more likely to die and therefore be underrepresented among long-term survivors. To explore this possibility, we examined the association between prior cognitive functioning and subsequent mortality and found that the inverse cognition-mortality association was actually stronger among controls than among cancer survivors. Although this finding does not support selective survival as the primary explanation for the better cognitive functioning observed among cancer survivors, survival bias cannot be completely ruled out. Accordingly, these findings should be interpreted cautiously and considered hypothesis-generating. Future studies designed to more directly address selective survival and informative attrition are needed. The inverse association between cancer diagnosis and Alzheimer’s and cognitive decline has been documented in previous studies [60–63]. In this study, we extended this line of evidence by minimizing pre-diagnosis differences using a matched design and provided long-term trajectories using population-based samples. In this study, the better cognitive functioning coincides with the higher quitting smoking rates among cancer survivors. Given that smoking is a known risk for cognitive impairment [64], smoking-related behavioral changes following a cancer diagnosis may partially explain the observed pattern. Other potential mechanisms include neuroplasticity and compensation for treatment-related damage, engagement with healthcare during cancer treatment, post-traumatic growth and hormetic effects. Future studies are needed to better understand these potential mechanisms.

### Limitations

Several limitations should be considered when interpreting these findings. First, the observational nature of the study precludes definitive causal inference, and residual confounding from unmeasured time-varying factors cannot be ruled out despite the matched design and fixed-effects DiD analyses. Second, because the HRS enrolls adults aged 50 years and older, findings may not generalize to individuals diagnosed with cancer at younger ages. Third, survivorship bias may have influenced the observed trajectories. Individuals with more aggressive cancers, greater treatment toxicity, poorer functional status, or worse cognitive functioning may have been more likely to die or drop out during follow-up and therefore be underrepresented among long-term survivors. Relatedly, informative attrition may have occurred if participants experiencing worse health outcomes were less likely to remain in the study, potentially leading to underestimation of the long-term burden associated with cancer diagnosis. Fourth, cancer status and most study outcomes were self-reported, introducing the potential for misclassification. Misclassification may be differential for some outcomes, particularly cognitive measures, because individuals with cognitive impairment may be less likely to accurately report a prior cancer diagnosis or other health conditions [65]. In addition, several lifestyle measures, including smoking, alcohol use, and physical activity, may be subject to social desirability and reporting biases. Fifth, the 5-year cutoff used to distinguish shorter-term and longer-term post-diagnosis periods in the DiD analyses was arbitrary. However, the primary trajectory analyses were conducted on a wave-by-wave basis, allowing a more granular assessment of changes over time. Thus, the trajectory plots and complementary DiD analyses jointly provide a more comprehensive characterization of long-term patterns across multiple domains before and after cancer diagnosis. Finally, the public-use HRS data lack detailed clinical information regarding cancer type, stage at diagnosis, treatment modality, treatment intensity, recurrence, and progression. Consequently, we were unable to assess heterogeneity across cancer characteristics or determine the extent to which the observed trajectories varied according to disease severity or treatment exposures. Given the substantial clinical diversity across cancers, the reported trajectories should therefore be interpreted as average patterns across a heterogeneous population of cancer survivors.

## Conclusion

In this population-based, longitudinal matched cohort study, we constructed long-term trajectories of health, behavioral, and economic outcomes before and after cancer diagnosis. Our findings reveal that cancer diagnosis is associated with profound and immediate disruptions across multiple domains, followed by persistent deficits in health, functioning, and financial well-being that can last for years. While some lifestyle changes, such as reductions in smoking, may indicate positive behavioral change, other gaps such as low physical activity and sustained economic burden highlight critical unmet needs in survivorship care.

## Supporting information

S1 Table Estimated outcome variables and differences at each time point. (DOCX)

S1 Fig Trajectories of vital status, health-related outcomes, Body Mass Index, health behaviors, work status, and out-of-pocket medical spending before and after cancer diagnosis among individuals diagnosed with incident cancer and their matched controls based on 1:1 match. Shaded area represents 95% confidence intervals.
(TIF)

S2 Fig Trajectories of vital status, health-related outcomes, Body Mass Index, health behaviors, work status, and out-of-pocket medical spending before and after cancer diagnosis among individuals diagnosed with incident cancer and their matched controls using matching weights and HRS weights. Shaded area represents 95% confidence intervals.
(DOCX)
