# Supplementary material for: Long-term comparative trajectories of health, behavioral, and economic outcomes: From pre-diagnosis to survivorship
Source: PLoS One. 2026 Sep 9;21(9):e0357917. doi: 10.1371/journal.pone.0357917 (PMC13557411; doi:10.1371/journal.pone.0357917)
Supplement: Supplementary file 1 [file pone.0357917.s003.docx]

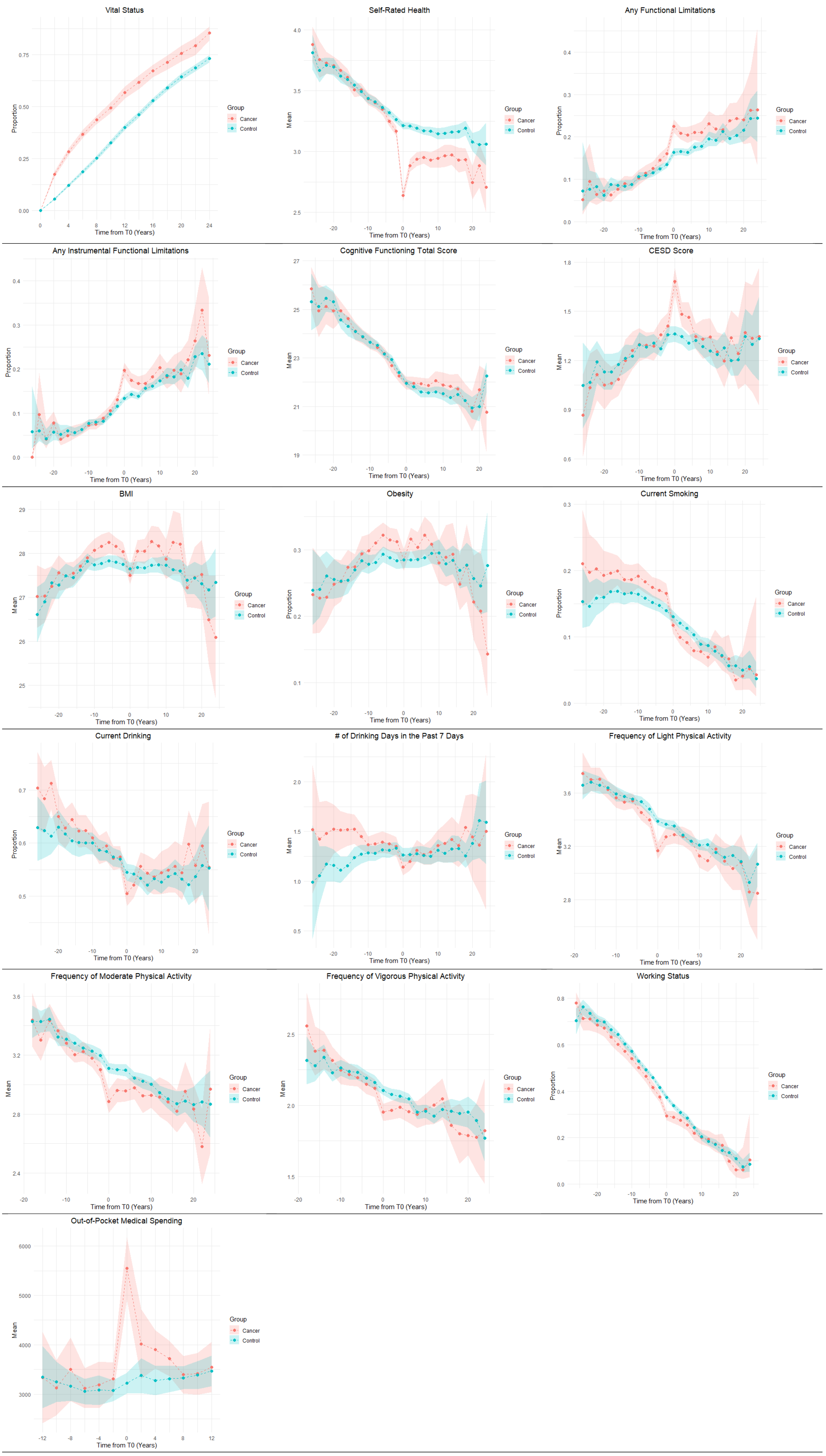
Vital Status
Self-Rated Health
Any Functional Limitations
Any Instrumental Functional Limitations
Cognitive Functioning Total Score
CESD Score
BMI
Obesity
Current Smoking
Current Drinking
# of Drinking Days in the Past 7 Days
Frequency of Light Physical Activity
Frequency of Moderate Physical Activity
Frequency of Vigorous Physical Activity
Working Status
Out-of-Pocket Medical Spending
Proportion
Mean
Time from T0 (Years)
Group
Cancer
Control
