# Supplementary material for: Long-term comparative trajectories of health, behavioral, and economic outcomes: From pre-diagnosis to survivorship
Source: PLoS One. 2026 Sep 9;21(9):e0357917. doi: 10.1371/journal.pone.0357917 (PMC13557411; doi:10.1371/journal.pone.0357917)
Supplement: Supplementary file 1 [file pone.0357917.s001.docx]

| S1 Table. Estimated outcome variables and differences at each time point | | | | |
| --- | --- | --- | --- | --- |
| Variable | Time | Mean or % - Case | Mean or % - Control | Difference |
| Death | 1 | 21% | 7% | 14% |
|  | 2 | 32% | 14% | 17% |
|  | 3 | 41% | 21% | 20% |
|  | 4 | 48% | 28% | 20% |
|  | 5 | 54% | 34% | 19% |
|  | 6 | 61% | 41% | 20% |
|  | 7 | 66% | 47% | 19% |
|  | 8 | 70% | 52% | 18% |
|  | 9 | 74% | 57% | 16% |
|  | 10 | 77% | 62% | 15% |
|  | 11 | 81% | 66% | 15% |
|  | 12 | 85% | 69% | 15% |
| Self-reported health | -12 | 3.70 | 3.65 | 0.04 |
|  | -11 | 3.69 | 3.64 | 0.05 |
|  | -10 | 3.68 | 3.61 | 0.07 |
|  | -9 | 3.61 | 3.59 | 0.01 |
|  | -8 | 3.57 | 3.56 | 0.01 |
|  | -7 | 3.46 | 3.52 | 0.06 |
|  | -6 | 3.43 | 3.46 | 0.03 |
|  | -5 | 3.38 | 3.40 | 0.02 |
|  | -4 | 3.34 | 3.36 | 0.02 |
|  | -3 | 3.29 | 3.31 | 0.02 |
|  | -2 | 3.20 | 3.26 | 0.06 |
|  | -1 | 3.10 | 3.19 | 0.09 |
|  | 0 | 2.54 | 3.12 | 0.58 |
|  | 1 | 2.78 | 3.11 | 0.33 |
|  | 2 | 2.83 | 3.09 | 0.26 |
|  | 3 | 2.86 | 3.07 | 0.20 |
|  | 4 | 2.86 | 3.06 | 0.20 |
|  | 5 | 2.87 | 3.04 | 0.17 |
|  | 6 | 2.88 | 3.05 | 0.17 |
|  | 7 | 2.88 | 3.06 | 0.18 |
|  | 8 | 2.82 | 3.06 | 0.24 |
|  | 9 | 2.83 | 3.09 | 0.25 |
|  | 10 | 2.72 | 3.02 | 0.30 |
|  | 11 | 2.80 | 2.96 | 0.16 |
|  | 12 | 2.70 | 2.98 | 0.28 |
| Any limitation in daily activities | -12 | 9% | 9% | 0% |
|  | -11 | 7% | 8% | 1% |
|  | -10 | 8% | 7% | 0% |
|  | -9 | 7% | 9% | 2% |
|  | -8 | 8% | 9% | 2% |
|  | -7 | 10% | 9% | 1% |
|  | -6 | 10% | 9% | 1% |
|  | -5 | 11% | 11% | 0% |
|  | -4 | 13% | 11% | 2% |
|  | -3 | 14% | 12% | 1% |
|  | -2 | 16% | 14% | 3% |
|  | -1 | 18% | 15% | 2% |
|  | 0 | 26% | 19% | 7% |
|  | 1 | 24% | 20% | 4% |
|  | 2 | 25% | 20% | 5% |
|  | 3 | 25% | 21% | 4% |
|  | 4 | 24% | 22% | 2% |
|  | 5 | 26% | 23% | 3% |
|  | 6 | 27% | 24% | 3% |
|  | 7 | 26% | 26% | 0% |
|  | 8 | 30% | 24% | 5% |
|  | 9 | 29% | 25% | 4% |
|  | 10 | 28% | 25% | 3% |
|  | 11 | 32% | 25% | 7% |
|  | 12 | 25% | 27% | 2% |
| Any limitation in instrumental daily activities | -12 | 7% | 6% | 0% |
|  | -11 | 4% | 4% | 0% |
|  | -10 | 8% | 6% | 2% |
|  | -9 | 5% | 6% | 1% |
|  | -8 | 5% | 6% | 1% |
|  | -7 | 6% | 6% | 0% |
|  | -6 | 7% | 7% | 0% |
|  | -5 | 8% | 8% | 0% |
|  | -4 | 9% | 8% | 0% |
|  | -3 | 10% | 9% | 1% |
|  | -2 | 12% | 11% | 1% |
|  | -1 | 15% | 14% | 1% |
|  | 0 | 24% | 17% | 7% |
|  | 1 | 21% | 17% | 3% |
|  | 2 | 20% | 18% | 2% |
|  | 3 | 20% | 20% | 1% |
|  | 4 | 22% | 20% | 2% |
|  | 5 | 24% | 21% | 2% |
|  | 6 | 23% | 23% | 0% |
|  | 7 | 24% | 23% | 1% |
|  | 8 | 26% | 24% | 1% |
|  | 9 | 27% | 24% | 4% |
|  | 10 | 29% | 26% | 3% |
|  | 11 | 37% | 25% | 12% |
|  | 12 | 30% | 26% | 3% |
| Cognitive functioning | -12 | 24.95 | 24.59 | 0.36 |
|  | -11 | 25.04 | 25.26 | 0.21 |
|  | -10 | 24.74 | 24.95 | 0.21 |
|  | -9 | 24.57 | 24.55 | 0.01 |
|  | -8 | 24.44 | 24.08 | 0.37 |
|  | -7 | 24.11 | 24.07 | 0.03 |
|  | -6 | 23.49 | 23.50 | 0.01 |
|  | -5 | 23.23 | 23.37 | 0.14 |
|  | -4 | 23.02 | 23.16 | 0.14 |
|  | -3 | 22.74 | 22.75 | 0.01 |
|  | -2 | 22.26 | 22.49 | 0.23 |
|  | -1 | 21.81 | 21.98 | 0.17 |
|  | 0 | 21.59 | 21.56 | 0.03 |
|  | 1 | 21.58 | 21.43 | 0.16 |
|  | 2 | 21.55 | 21.13 | 0.43 |
|  | 3 | 21.45 | 21.07 | 0.38 |
|  | 4 | 21.39 | 21.03 | 0.36 |
|  | 5 | 21.33 | 21.02 | 0.32 |
|  | 6 | 21.20 | 20.92 | 0.28 |
|  | 7 | 21.08 | 20.96 | 0.12 |
|  | 8 | 20.95 | 20.69 | 0.26 |
|  | 9 | 20.51 | 20.47 | 0.04 |
|  | 10 | 21.51 | 20.48 | 1.04 |
|  | 11 | 20.92 | 22.02 | -1.10 |
| CESD | -12 | 1.11 | 1.12 | 0.02 |
|  | -11 | 1.17 | 1.28 | 0.11 |
|  | -10 | 1.11 | 1.24 | 0.13 |
|  | -9 | 1.15 | 1.20 | 0.04 |
|  | -8 | 1.12 | 1.22 | 0.10 |
|  | -7 | 1.25 | 1.22 | 0.03 |
|  | -6 | 1.32 | 1.26 | 0.06 |
|  | -5 | 1.33 | 1.29 | 0.05 |
|  | -4 | 1.33 | 1.30 | 0.03 |
|  | -3 | 1.35 | 1.34 | 0.01 |
|  | -2 | 1.40 | 1.34 | 0.07 |
|  | -1 | 1.46 | 1.42 | 0.05 |
|  | 0 | 1.76 | 1.43 | 0.33 |
|  | 1 | 1.57 | 1.43 | 0.14 |
|  | 2 | 1.54 | 1.41 | 0.12 |
|  | 3 | 1.46 | 1.39 | 0.06 |
|  | 4 | 1.45 | 1.36 | 0.09 |
|  | 5 | 1.44 | 1.33 | 0.11 |
|  | 6 | 1.39 | 1.31 | 0.08 |
|  | 7 | 1.30 | 1.35 | 0.05 |
|  | 8 | 1.36 | 1.30 | 0.06 |
|  | 9 | 1.37 | 1.28 | 0.10 |
|  | 10 | 1.49 | 1.36 | 0.13 |
|  | 11 | 1.47 | 1.32 | 0.15 |
|  | 12 | 1.56 | 1.37 | 0.19 |
| BMI | -12 | 27.14 | 26.91 | 0.23 |
|  | -11 | 27.19 | 27.20 | 0.01 |
|  | -10 | 27.44 | 27.24 | 0.20 |
|  | -9 | 27.45 | 27.29 | 0.16 |
|  | -8 | 27.56 | 27.36 | 0.20 |
|  | -7 | 27.62 | 27.46 | 0.16 |
|  | -6 | 27.82 | 27.66 | 0.16 |
|  | -5 | 27.83 | 27.63 | 0.19 |
|  | -4 | 27.90 | 27.65 | 0.25 |
|  | -3 | 27.96 | 27.70 | 0.26 |
|  | -2 | 27.90 | 27.66 | 0.23 |
|  | -1 | 27.76 | 27.60 | 0.16 |
|  | 0 | 27.19 | 27.52 | -0.33 |
|  | 1 | 27.80 | 27.54 | 0.27 |
|  | 2 | 27.86 | 27.57 | 0.28 |
|  | 3 | 27.92 | 27.54 | 0.39 |
|  | 4 | 27.92 | 27.54 | 0.38 |
|  | 5 | 27.66 | 27.55 | 0.10 |
|  | 6 | 27.92 | 27.41 | 0.51 |
|  | 7 | 27.90 | 27.38 | 0.52 |
|  | 8 | 27.41 | 27.21 | 0.20 |
|  | 9 | 27.44 | 27.20 | 0.24 |
|  | 10 | 27.02 | 27.20 | 0.19 |
|  | 11 | 26.86 | 27.06 | 0.20 |
|  | 12 | 26.35 | 27.05 | 0.71 |
| Obesity | -12 | 25% | 24% | 1% |
|  | -11 | 23% | 25% | -2% |
|  | -10 | 26% | 25% | 0% |
|  | -9 | 25% | 25% | 0% |
|  | -8 | 27% | 25% | 1% |
|  | -7 | 27% | 26% | 1% |
|  | -6 | 29% | 27% | 1% |
|  | -5 | 28% | 27% | 1% |
|  | -4 | 29% | 27% | 1% |
|  | -3 | 30% | 28% | 2% |
|  | -2 | 29% | 28% | 1% |
|  | -1 | 29% | 28% | 1% |
|  | 0 | 26% | 28% | -1% |
|  | 1 | 30% | 28% | 2% |
|  | 2 | 30% | 28% | 1% |
|  | 3 | 30% | 28% | 2% |
|  | 4 | 30% | 28% | 2% |
|  | 5 | 28% | 28% | -1% |
|  | 6 | 28% | 27% | 1% |
|  | 7 | 29% | 27% | 1% |
|  | 8 | 26% | 27% | -1% |
|  | 9 | 27% | 27% | 0% |
|  | 10 | 24% | 27% | -3% |
|  | 11 | 23% | 24% | -1% |
|  | 12 | 18% | 24% | -6% |
| Current smoking | -12 | 21% | 16% | 4% |
|  | -11 | 20% | 17% | 3% |
|  | -10 | 19% | 18% | 1% |
|  | -9 | 20% | 19% | 1% |
|  | -8 | 21% | 18% | 2% |
|  | -7 | 19% | 17% | 2% |
|  | -6 | 19% | 17% | 2% |
|  | -5 | 20% | 17% | 3% |
|  | -4 | 19% | 16% | 2% |
|  | -3 | 18% | 16% | 2% |
|  | -2 | 17% | 15% | 2% |
|  | -1 | 17% | 15% | 2% |
|  | 0 | 11% | 13% | -2% |
|  | 1 | 10% | 12% | -2% |
|  | 2 | 9% | 11% | -2% |
|  | 3 | 9% | 10% | -2% |
|  | 4 | 8% | 9% | -1% |
|  | 5 | 7% | 8% | -1% |
|  | 6 | 8% | 8% | 0% |
|  | 7 | 6% | 7% | -1% |
|  | 8 | 6% | 7% | 0% |
|  | 9 | 5% | 6% | -1% |
|  | 10 | 5% | 5% | 0% |
|  | 11 | 5% | 5% | 0% |
|  | 12 | 5% | 4% | 0% |
| Current drinking | -12 | 64% | 60% | 4% |
|  | -11 | 65% | 57% | 8% |
|  | -10 | 61% | 58% | 3% |
|  | -9 | 61% | 57% | 4% |
|  | -8 | 62% | 57% | 5% |
|  | -7 | 59% | 57% | 2% |
|  | -6 | 58% | 56% | 2% |
|  | -5 | 58% | 56% | 1% |
|  | -4 | 57% | 55% | 2% |
|  | -3 | 56% | 55% | 1% |
|  | -2 | 54% | 54% | 1% |
|  | -1 | 54% | 53% | 1% |
|  | 0 | 47% | 50% | -3% |
|  | 1 | 49% | 49% | -1% |
|  | 2 | 51% | 49% | 2% |
|  | 3 | 51% | 48% | 3% |
|  | 4 | 50% | 49% | 2% |
|  | 5 | 50% | 48% | 2% |
|  | 6 | 49% | 49% | 1% |
|  | 7 | 51% | 49% | 1% |
|  | 8 | 51% | 49% | 2% |
|  | 9 | 54% | 49% | 5% |
|  | 10 | 52% | 49% | 3% |
|  | 11 | 53% | 50% | 2% |
|  | 12 | 54% | 49% | 5% |
| # of drinking days in past 7 days | -12 | 1.31 | 1.03 | 0.28 |
|  | -11 | 1.34 | 1.05 | 0.29 |
|  | -10 | 1.37 | 1.01 | 0.36 |
|  | -9 | 1.34 | 1.03 | 0.31 |
|  | -8 | 1.33 | 1.07 | 0.27 |
|  | -7 | 1.39 | 1.14 | 0.25 |
|  | -6 | 1.30 | 1.15 | 0.15 |
|  | -5 | 1.29 | 1.15 | 0.15 |
|  | -4 | 1.30 | 1.16 | 0.14 |
|  | -3 | 1.30 | 1.17 | 0.13 |
|  | -2 | 1.28 | 1.18 | 0.09 |
|  | -1 | 1.26 | 1.18 | 0.08 |
|  | 0 | 1.04 | 1.12 | -0.08 |
|  | 1 | 1.11 | 1.12 | -0.01 |
|  | 2 | 1.17 | 1.12 | 0.05 |
|  | 3 | 1.15 | 1.11 | 0.04 |
|  | 4 | 1.14 | 1.13 | 0.01 |
|  | 5 | 1.18 | 1.15 | 0.03 |
|  | 6 | 1.18 | 1.13 | 0.04 |
|  | 7 | 1.19 | 1.15 | 0.05 |
|  | 8 | 1.22 | 1.14 | 0.08 |
|  | 9 | 1.29 | 1.16 | 0.13 |
|  | 10 | 1.33 | 1.18 | 0.15 |
|  | 11 | 1.39 | 1.30 | 0.08 |
|  | 12 | 1.34 | 1.30 | 0.03 |
| # of drinks on days drank | -12 | 0.80 | 0.75 | 0.06 |
|  | -11 | 0.86 | 0.67 | 0.19 |
|  | -10 | 0.89 | 0.69 | 0.20 |
|  | -9 | 0.80 | 0.70 | 0.09 |
|  | -8 | 0.82 | 0.75 | 0.07 |
|  | -7 | 0.86 | 0.76 | 0.11 |
|  | -6 | 0.84 | 0.76 | 0.08 |
|  | -5 | 0.84 | 0.76 | 0.09 |
|  | -4 | 0.85 | 0.77 | 0.08 |
|  | -3 | 0.82 | 0.76 | 0.06 |
|  | -2 | 0.77 | 0.76 | 0.01 |
|  | -1 | 0.78 | 0.75 | 0.04 |
|  | 0 | 0.60 | 0.68 | -0.09 |
|  | 1 | 0.61 | 0.66 | -0.05 |
|  | 2 | 0.67 | 0.64 | 0.03 |
|  | 3 | 0.63 | 0.60 | 0.02 |
|  | 4 | 0.64 | 0.60 | 0.04 |
|  | 5 | 0.63 | 0.60 | 0.03 |
|  | 6 | 0.58 | 0.59 | 0.02 |
|  | 7 | 0.60 | 0.57 | 0.03 |
|  | 8 | 0.59 | 0.57 | 0.02 |
|  | 9 | 0.59 | 0.55 | 0.05 |
|  | 10 | 0.63 | 0.51 | 0.12 |
|  | 11 | 0.62 | 0.60 | 0.02 |
|  | 12 | 0.58 | 0.52 | 0.06 |
| Frequency of light activities | -9 | 3.74 | 3.65 | 0.09 |
|  | -8 | 3.67 | 3.68 | -0.01 |
|  | -7 | 3.69 | 3.65 | 0.04 |
|  | -6 | 3.61 | 3.61 | 0.00 |
|  | -5 | 3.54 | 3.57 | -0.03 |
|  | -4 | 3.48 | 3.52 | -0.04 |
|  | -3 | 3.47 | 3.49 | -0.02 |
|  | -2 | 3.35 | 3.46 | -0.11 |
|  | -1 | 3.29 | 3.39 | -0.09 |
|  | 0 | 3.03 | 3.27 | -0.24 |
|  | 1 | 3.15 | 3.24 | -0.09 |
|  | 2 | 3.14 | 3.20 | -0.06 |
|  | 3 | 3.13 | 3.13 | 0.00 |
|  | 4 | 3.10 | 3.09 | 0.00 |
|  | 5 | 3.00 | 3.05 | -0.05 |
|  | 6 | 2.99 | 3.06 | -0.07 |
|  | 7 | 2.99 | 3.02 | -0.03 |
|  | 8 | 2.93 | 2.98 | -0.05 |
|  | 9 | 2.88 | 2.97 | -0.10 |
|  | 10 | 2.92 | 3.02 | -0.09 |
|  | 11 | 2.81 | 2.92 | -0.11 |
|  | 12 | 2.78 | 2.99 | -0.21 |
| Frequency of moderate activities | -9 | 3.38 | 3.41 | -0.02 |
|  | -8 | 3.28 | 3.41 | -0.13 |
|  | -7 | 3.35 | 3.39 | -0.03 |
|  | -6 | 3.33 | 3.29 | 0.04 |
|  | -5 | 3.24 | 3.27 | -0.03 |
|  | -4 | 3.14 | 3.22 | -0.08 |
|  | -3 | 3.15 | 3.19 | -0.04 |
|  | -2 | 3.06 | 3.13 | -0.07 |
|  | -1 | 2.98 | 3.08 | -0.10 |
|  | 0 | 2.74 | 2.99 | -0.25 |
|  | 1 | 2.84 | 2.99 | -0.15 |
|  | 2 | 2.81 | 2.96 | -0.14 |
|  | 3 | 2.86 | 2.91 | -0.05 |
|  | 4 | 2.81 | 2.90 | -0.09 |
|  | 5 | 2.82 | 2.86 | -0.05 |
|  | 6 | 2.77 | 2.80 | -0.03 |
|  | 7 | 2.69 | 2.79 | -0.10 |
|  | 8 | 2.65 | 2.75 | -0.10 |
|  | 9 | 2.75 | 2.77 | -0.01 |
|  | 10 | 2.70 | 2.76 | -0.06 |
|  | 11 | 2.50 | 2.80 | -0.29 |
|  | 12 | 2.85 | 2.79 | 0.06 |
| Frequency of vigorous activities | -9 | 2.41 | 2.22 | 0.18 |
|  | -8 | 2.29 | 2.22 | 0.07 |
|  | -7 | 2.30 | 2.27 | 0.04 |
|  | -6 | 2.26 | 2.19 | 0.07 |
|  | -5 | 2.19 | 2.20 | -0.01 |
|  | -4 | 2.15 | 2.15 | 0.00 |
|  | -3 | 2.13 | 2.13 | 0.00 |
|  | -2 | 2.08 | 2.10 | -0.02 |
|  | -1 | 2.01 | 2.07 | -0.06 |
|  | 0 | 1.82 | 2.01 | -0.19 |
|  | 1 | 1.86 | 1.97 | -0.11 |
|  | 2 | 1.87 | 1.97 | -0.10 |
|  | 3 | 1.85 | 1.94 | -0.09 |
|  | 4 | 1.88 | 1.88 | 0.00 |
|  | 5 | 1.88 | 1.87 | 0.01 |
|  | 6 | 1.91 | 1.83 | 0.08 |
|  | 7 | 1.93 | 1.87 | 0.05 |
|  | 8 | 1.77 | 1.88 | -0.11 |
|  | 9 | 1.78 | 1.87 | -0.08 |
|  | 10 | 1.78 | 1.87 | -0.09 |
|  | 11 | 1.81 | 1.87 | -0.07 |
|  | 12 | 1.85 | 1.78 | 0.08 |
| Employed | -12 | 70% | 75% | -4% |
|  | -11 | 68% | 70% | -2% |
|  | -10 | 65% | 68% | -2% |
|  | -9 | 64% | 66% | -2% |
|  | -8 | 61% | 63% | -2% |
|  | -7 | 56% | 61% | -3% |
|  | -6 | 53% | 56% | -3% |
|  | -5 | 51% | 54% | -3% |
|  | -4 | 47% | 49% | -3% |
|  | -3 | 43% | 46% | -3% |
|  | -2 | 38% | 42% | -4% |
|  | -1 | 33% | 37% | -4% |
|  | 0 | 25% | 33% | -8% |
|  | 1 | 24% | 30% | -6% |
|  | 2 | 23% | 27% | -4% |
|  | 3 | 21% | 24% | -3% |
|  | 4 | 18% | 21% | -3% |
|  | 5 | 17% | 18% | -1% |
|  | 6 | 16% | 16% | -1% |
|  | 7 | 15% | 15% | 0% |
|  | 8 | 13% | 13% | 1% |
|  | 9 | 9% | 12% | -4% |
|  | 10 | 7% | 9% | -2% |
|  | 11 | 7% | 9% | -2% |
|  | 12 | 7% | 6% | 1% |
| Out-of-pocket medical expenses ($) | -6 | 3412 | 2799 | 614 |
|  | -5 | 3029 | 2885 | 144 |
|  | -4 | 3213 | 3042 | 172 |
|  | -3 | 2951 | 2915 | 36 |
|  | -2 | 3301 | 3092 | 210 |
|  | -1 | 3355 | 3078 | 277 |
|  | 0 | 5779 | 3397 | 2382 |
|  | 1 | 4091 | 3416 | 676 |
|  | 2 | 4106 | 3530 | 577 |
|  | 3 | 4443 | 3796 | 647 |
|  | 4 | 4208 | 3830 | 378 |
|  | 5 | 4097 | 3928 | 168 |
|  | 6 | 4336 | 4301 | 35 |
|  | 7 | 4494 | 4102 | 392 |
|  | 8 | 4321 | 4002 | 319 |
|  | 9 | 6566 | 4825 | 1741 |
|  | 10 | 4047 | 3968 | 78 |
|  | 11 | 3991 | 4011 | -19 |
|  | 12 | 4091 | 3914 | 177 |
