# Supplementary material for: Long-term comparative trajectories of health, behavioral, and economic outcomes: From pre-diagnosis to survivorship
Source: PLoS One. 2026 Sep 9;21(9):e0357917. doi: 10.1371/journal.pone.0357917 (PMC13557411; doi:10.1371/journal.pone.0357917)
Supplement: Supplementary file 3 [file pone.0357917.s004.docx]

**Long-Term Comparative Trajectories of Health, Behavioral, and Economic Outcomes:**

**From Pre-Diagnosis to Survivorship**

Overall Evaluation of the Manuscript

This manuscript presents a longitudinal matched cohort study examining long-term trajectories of health, behavioral, and economic outcomes before and after cancer diagnosis using data from the Health and Retirement Study (HRS). The topic is highly relevant and addresses an important gap in cancer survivorship research by adopting a multidimensional and long-term perspective. The use of a large, nationally representative dataset and the attempt to align trajectories around the time of diagnosis represent notable strengths. However, despite its potential contribution, the manuscript raises several important methodological, analytical, and interpretative concerns that need to be addressed to meet the standards of a high-impact scientific journal. The following review provides a detailed critical appraisal of each section of the manuscript, highlighting key limitations and offering specific recommendations for improvement.

I. **TITLE & ABSTRACT**

**Major Issues**

- The title is too long and insufficiently specific:
  - Does not clearly indicate the study design (matched cohort)
  - Does not specify the population (≥50 years, USA)
- The abstract is mainly descriptive, lacking analytical depth:
  - No mention of statistical models
  - No reporting of effect sizes (β, OR, HR)

**Comments**

- Results are presented as percentages → insufficient for a high-impact journal
- Ambiguity between association vs causation

**Recommendations**

- Revise title:
   “A longitudinal matched cohort study of multidimensional trajectories before and after cancer diagnosis among US adults aged ≥50 (HRS 1992–2022)”
- Improve abstract:
  - Specify statistical methods (e.g., mixed models, weighted analysis)
  - Include key effect estimates
  - Clearly state hypothesis

II. **INTRODUCTION**

**Major Issues**

- The introduction is descriptive rather than analytical
- No explicit research hypothesis
- Lack of a conceptual/theoretical framework

**Comments**

- Good justification of research gap
- However, lacks:
  - Structured argumentation
  - Prioritization of gaps

**recommendations**

- Add:
  - A clear research question
  - Explicit testable hypotheses
  - A conceptual framework (e.g., survivorship model, biopsychosocial model)

III. **METHODS**

Major Limitations

**1. Residual Confounding**

- Matching limited to:
  - Sex, age, race, entry wave
- Missing critical confounders:
  1. Comorbidities
  2. Socioeconomic status
  3. Baseline health conditions

- High risk of residual confounding.

**2. Inadequate Statistical Approach**

- Analysis limited to:
  - Means/proportions + confidence intervals
- No advanced longitudinal modeling:
  - Mixed-effects models
  - GEE (Generalized Estimating Equation)
  - Trajectory modeling

-This is a **major methodological weakness**

**3. Causal Interpretation Risk**

- Observational design but interpreted partially as causal

**4. Cancer as a Single Entity**

- No stratification by:
  - Cancer type
  - Stage
  - Treatment

-Limits clinical relevance.

**5. Measurement Bias**

- Self-reported cancer and behaviors → risk of misclassification

**Comments**

- Strong dataset (HRS)
- Matching strategy appropriate
- Sensitivity analyses included

**Recommendations**

**Statistical Improvements**

Use:

1. Linear mixed models
2. Generalized estimating equations (GEE)
3. Growth curve modeling

**Adjustments**

- Include:
  1. Comorbidities
  2. Income/education
  3. Insurance status

**Additional Analyses**

- Stratified analyses:
  - Age, Sex
  - Cancer type (if possible)
- Survival analysis (Cox regression)

IV. **RESULTS**

**Major Issues**

- Results are too descriptive
- Missing:
  1. Effect sizes
  2. Regression outputs

**Comments**

- Figures (pages 27–29) are clear and informative
- Good visualization of trajectories
- detailed p-values

**Recommendations**

Add:

1. Multivariable regression tables
2. Adjusted estimates
3. Interaction analyses

V. **DISCUSSION**

**Major Issues**

- Overinterpretation of findings
- Some conclusions imply causality
- Cognitive findings are highly speculative

**Comments**

1. Good linkage with literature
2. Rich but sometimes narrative

**Recommendations**

- Clearly separate:
  1. Confirmed findings
  2. Hypotheses/speculation
- Reduce unsupported biological interpretations
- Strengthen discussion on bias:
  1. Survivorship bias ;2. Attrition bias

VI. **LIMITATIONS**

Missing Critical Limitations

Current limitations are insufficient. Missing:

- Selection bias
- Attrition bias (longitudinal study)
- Survivorship bias
- Residual confounding
- Lack of clinical detail (cancer type, stage)

**Recommendations**

1. Expand limitations section significantly
2. Explicitly state:
   “ Residual confounding cannot be excluded”
VII. **CONCLUSION**

**Issues**

- Too general
- Limited practical implications

**Recommendations**

Add:

- Clinical implications
- Policy implications
- Future research directions

VIII. **REFERENCES**

**Issues**

- Inconsistent referencing style (not strictly Vancouver/AMA)
- Some non-academic sources (e.g., SlideShare)
- Formatting inconsistencies

**Comments**

- References are recent and relevant overall

**recommendations**

1. Standardize referencing style
2. Remove weak sources
3. Ensure all references include DOI
IX. **WRITING STYLE**

**Issues**

- Some sentences are:
  1. Too long
  2. Repetitive
- Occasionally narrative rather than analytical

**comments**

- Overall good academic English

**recommendations**

- Shorten sentences
- Use more precise scientific language
- Avoid overstatements

X. **OVERALL ASSESSMENT**

Overall, this manuscript addresses an important and timely topic in cancer survivorship by exploring long-term multidimensional trajectories using a large, nationally representative dataset. The longitudinal design, extended follow-up period, and matched cohort approach constitute notable strengths that enhance the potential scientific contribution of the study. However, the overall methodological rigor remains limited, particularly due to the absence of advanced longitudinal statistical modeling and insufficient adjustment for key confounding variables such as comorbidities and socioeconomic status. The analysis is predominantly descriptive, which restricts the interpretability and robustness of the findings. In addition, the manuscript occasionally overinterprets associations as causal relationships, which is not justified given the observational design. Important sources of bias, including survivorship bias, attrition bias, and residual confounding, are not adequately discussed. The lack of clinical granularity, particularly regarding cancer type and treatment, further limits the applicability of the results. While the discussion is comprehensive, it includes speculative explanations that are not directly supported by the data. The referencing style also requires standardization to meet journal requirements. Overall, substantial revisions are needed to strengthen the methodological, analytical, and interpretative aspects of the manuscript before it can be considered for publication.
