# Supplementary material for: Long-term comparative trajectories of health, behavioral, and economic outcomes: From pre-diagnosis to survivorship
Source: PLoS One. 2026 Sep 9;21(9):e0357917. doi: 10.1371/journal.pone.0357917 (PMC13557411; doi:10.1371/journal.pone.0357917)
Supplement: Supplementary file 5 [file pone.0357917.s006.docx]

**Manuscript title:** Long-Term Comparative Trajectories of Health, Behavioral, and Economic Outcomes: From Pre-Diagnosis to Survivorship

**Manuscript ID:** PONE-D-25-60630

**Reviewer #1**

1. This manuscript addresses an important and timely topic in cancer survivorship, examining the long-term trajectories of health, behavioural and economic outcomes using a large nationally representative longitudinal dataset (the Health and Retirement Study, HRS). The study design, which is based on a matched cohort approach and an extended follow-up period, is a clear strength that provides valuable insights into multidimensional survivorship patterns over time. The manuscript is well structured and written in standard academic English, and the topic is clearly relevant to public health and policy.

Response: We thank the reviewer for the thoughtful assessment of our study and for recognizing the significance and strengths of the study. We appreciate the constructive comments and suggestions from the reviewer, and we have revised the manuscript accordingly. Our detailed responses to each comment are presented below.

2. However, there are several important methodological and analytical issues that need to be addressed. Firstly, the statistical analysis is largely descriptive and does not make full use of the longitudinal nature of the data. The absence of advanced modelling approaches, such as mixed-effects models or Generalised Estimating Equations (GEE), limits the robustness and interpretability of the findings.

Response: We thank the reviewer for this comment and agree that adding inferential statistics would significantly enhance the study. During the revision, we have conducted difference-in-difference (DiD) models to compare changes in outcomes at diagnosis and post- diagnosis waves. Specifically, we estimated a two-way fixed-effects DiD model for each outcome:

where Y_it_ represents the outcome for individua *i* at survey wave *t*; Cancer_i_ indicates group (case or control); Period_kt_ denotes time periods relative to diagnosis (pre-diagnosis, diagnosis wave, 1-5 years post-diagnosis, and >5 years post-diagnosis); α_i_ represents individual fixed effects; and γ_t_ represents time-period fixed effects. Standard errors were two-way clustered on matched pair and each participant to account for both the matched design and within-person correlation across repeated waves.

We note that although mixed-effects models and GEE are widely used for longitudinal data, our primary objective was to estimate how outcomes changed following cancer diagnosis relative to contemporaneous changes among matched controls. This comparative longitudinal contrast is the central focus of a DiD framework. The fixed-effects DiD approach explicitly leverages the repeated-measures structure of the data by comparing within-person changes over time while controlling for stable individual characteristics, thereby reducing bias from time-invariant confounding. Moreover, the resulting interaction terms can be interpreted directly as the differential change in outcomes among cancer survivors relative to controls at each stage following diagnosis, which aligns closely with our study aims.

In addition, the fixed-effects DiD holds constant all observed and unobserved time-invariant characteristics (e.g., genetic make-up), so it requires weaker assumptions than a mixed-effects model regarding the relationship between individual-specific effects and observed covariates. In particular, fixed-effects models allow person-level characteristics to be correlated with cancer incidence and subsequent outcomes, whereas standard random-effects specifications require these effects to be independent of model covariates. Given the possibility that unobserved baseline health status and long-term health trajectories may be associated with both cancer risk and study outcomes, we considered the fixed-effects DiD framework to be the more appropriate analytic approach.

To further strengthen the longitudinal rigor of the analysis, we additionally conducted (1) a formal test of pre-diagnosis parallel trends using wave-specific treatment interactions restricted to the pre-diagnosis period and (2) false discovery rate (Benjamini-Hochberg) adjustment to account for multiple comparisons across the 15 outcomes examined. We have added a Table 2 to present the results and described it in the revised Methods and Results section.

Taken together, we believe these revisions substantially strengthen the inferential component of the study while providing a longitudinal analytical framework that is closely aligned with the study's objective of quantifying changes associated with cancer diagnosis relative to matched controls.

3. Secondly, although the matching strategy is appropriate, it does not account for key potential confounders such as comorbidities, socioeconomic status or baseline health conditions. This raises concerns about residual confounding.

Response: We thank the reviewer for raising this important concern. We agree that residual confounding is a consideration in any observational study and cannot be completely eliminated. Our matching strategy was intentionally limited to sex, age, race/ethnicity, and study entry wave for several reasons.

First, the primary objective of this study was to characterize and compare long-term trajectories of health, behavioral, and economic outcomes among individuals with incident cancer and matched controls, rather than to estimate a causal effect of cancer diagnosis. Given the broad range of outcomes examined, there is no single set of covariates that would serve as confounders for all outcomes. For example, factors that may confound behavioral outcomes may differ substantially from those relevant to economic or health outcomes. Consequently, matching on an extensive set of variables would be challenging to justify across all analyses.

Second, matching on factors strongly associated with cancer risk, such as comorbidity burden and socioeconomic status, could substantially alter the underlying risk profile of the comparison group and may reduce the representativeness of controls drawn from the source population. Instead, we sought to identify controls with similar demographic characteristics and follow-up structure while preserving the natural differences in cancer risk profiles between groups.

Importantly, examination of the pre-diagnosis trajectories showed that most outcomes exhibited very similar levels and trends between cancer survivors and controls prior to diagnosis, including self-rated health. Consistent with existing knowledge about risk factors of cancer, we observed higher BMI, higher prevalence of smoking and alcohol use, and lower prevalence of physical activity during the pre-diagnosis period among the case group; however, the trajectories of these variables were generally parallel before diagnosis, suggesting that subsequent divergences were unlikely to be driven solely by differential pre-diagnosis trends.

In response to the reviewer's concern, we have strengthened the analysis by incorporating a fixed-effects difference-in-differences (DiD) framework. This approach controls for all observed and unobserved time-invariant individual characteristics, including stable aspects of socioeconomic status and comorbidity burden, thereby reducing confounding from pre-existing differences between cancer survivors and controls. Nevertheless, we acknowledge that time-varying factors, including changes in comorbidity burden over time, cannot be fully ruled out and that causal inference is limited by the observational nature of the study. This limitation is clarified in the manuscript. We have also revised the study aims to emphasize the descriptive and comparative nature of the analyses.

4. Thirdly, the observational design precludes causal inference; however, some of the interpretations in the manuscript appear to use causal language and should therefore be revised.

Response: We thank the reviewer for raising this concern. We have thoroughly reviewed the text and removed such casual language.

5. Additionally, key sources of bias, such as survivorship, attrition, and measurement biases related to self-reported data, are not adequately addressed in the limitations section. The lack of clinical granularity, particularly with regard to cancer type, stage and treatment modalities, further limits the applicability and interpretability of the findings.

Response: We thank the reviewer for highlighting these important issues. We agree that survivorship bias, attrition, measurement error, and limited clinical detail are important considerations in interpreting our findings. In response, we have substantially expanded the Limitations section to explicitly discuss survivorship bias, informative attrition, measurement error and misclassification associated with self-reported cancer status and behavioral outcomes, and the absence of detailed clinical information on cancer type, stage, recurrence, and treatment modalities in the public HRS dataset. We now acknowledge that these factors may influence the estimated trajectories and limit our ability to evaluate heterogeneity across cancer subgroups. These additions are included in the revised Discussion.

6. The results section would benefit from more detailed statistical reporting, including effect sizes and multivariable analyses.

Response: We thank the reviewer for this valuable suggestion. In response, we have expanded the Results section to provide more detailed statistical reporting. Specifically, we now present quantitative estimates with 95% confidence intervals from the wave-by-wave descriptive analyses. In addition, we incorporated multivariable longitudinal analyses using a two-way fixed-effects difference-in-differences (DiD) framework to compare changes in outcomes at the wave of diagnosis, within 5 years after diagnosis, and more than 5 years after diagnosis. The DiD approach accounts for individual-level time-invariant characteristics as well as secular time trends, thereby providing more robust estimates of the association between cancer diagnosis and subsequent changes in outcomes. The Results section has been revised to report these model estimates alongside the descriptive trajectories. Together, the descriptive and multivariable findings allow readers to better evaluate the magnitude, precision, and statistical significance of the observed associations.

7. While the discussion is comprehensive, it includes some speculative explanations that are not directly supported by the data and should be tempered.

Response: We thank the reviewer for this helpful observation. We have carefully reviewed the Discussion and revised the language to better distinguish empirical findings from potential explanations. Specifically, we have tempered causal and mechanistic interpretations, emphasized the observational nature of the study, and framed proposed explanations as hypotheses that warrant further investigation. We have also added qualifying language (e.g., “may,” “might,” and “could”) where appropriate and clarified that these explanations are intended to provide context.

8. In terms of transparency, the data availability statement is appropriate given that the HRS dataset is publicly accessible. However, providing additional details about dataset versions and analytical procedures would strengthen reproducibility further. While the reference list is generally relevant and up to date, improvements in formatting consistency and the removal of non-academic sources are recommended.

Response: We appreciate this thoughtful suggestion. We have revised the Data Availability section to specify the exact data file used. We have also expanded the Methods section to include more detailed descriptions of the cohort and analytical approaches. Additionally, we have reviewed the reference list and removed non-academic sources where appropriate.

9. No major concerns relating to research ethics, dual publication or publication ethics were identified. Overall, the manuscript shows strong potential, but substantial revisions are required to improve its methodological rigour, analytical depth and clarity of interpretation before it can be considered for publication.

Response: We thank the reviewer for the thoughtful and constructive assessment of our manuscript and for recognizing its potential contribution to the literature on cancer survivorship. We appreciate the comments regarding the need to strengthen methodological rigor, enhance analytical depth, and improve clarity of interpretation. In response, we have undertaken substantial revisions to address these points (see our responses above). We believe that these revisions substantially improve the methodological rigor, analytical clarity, and overall quality of the manuscript, and we are grateful to the reviewer for the guidance that led to these improvements.

**Reviewer #2**

This study uses a large, well-established longitudinal dataset to examine health trajectories before and after cancer diagnosis across multiple domains. The long follow-up period, matched-cohort design, and broad range of outcomes are notable strengths. However, several methodological and interpretive concerns should be addressed before the manuscript is suitable for publication.

Response: We thank the reviewer for the thoughtful assessment of our study and for recognizing its key strengths. We appreciate the constructive comments and suggestions for improvement. We have carefully considered each concern, revised the manuscript accordingly, and provide detailed point-by-point responses below.

Major Concerns

1. The reported finding of better cognitive function among cancer survivors may be influenced by survivor bias. Patients with cancer who have poorer cognition may be more likely to die and leave the analysis, resulting in a progressively healthier surviving cohort over time. The comparison of cognition–mortality associations between groups does not fully address this concern. Please discuss the potential impact of differential survival on the cognitive findings and consider additional analyses (e.g. sensitivity analyses) to assess the robustness of this result. At minimum, the cognitive findings should be interpreted more cautiously and framed as hypothesis-generating.

Response: We thank the reviewer for this important comment. We agree that selective survival is a potential concern in studies of aging and cancer survivorship and could influence estimates of cognitive functioning among survivors. To address this issue, we conducted an additional analysis examining whether the association between cognitive functioning at the prior wave and subsequent mortality differed between cancer survivors and controls (last two sentences in the 3^rd^ paragraph of Results): “To assess the potential role of survival, we used a mixed-effects model to estimate group differences in the association between cognitive function at the prior wave and death at the current wave. The results showed a stronger inverse association among controls, with ORs of 0.89 (95% CI=0.88, 0.89) and 0.92 (95% CI=0.91, 0.94) in the control and cancer groups, respectively (p < 0.001 for the interaction term).” These findings suggest that differential mortality related to cognitive functioning is more pronounced in the control group and therefore suggests that differential mortality related to cognitive functioning is unlikely to fully explain the higher cognitive functioning observed among cancer survivors.

Nevertheless, we agree that survivor bias cannot be completely excluded, and we have revised the Discussion to acknowledge this limitation and to interpret the cognitive findings more cautiously. We now explicitly note that the observed association should be considered hypothesis-generating and that future studies using methods specifically designed to account for informative dropout and selective survival are needed to confirm these findings.

2. A large number of repeated paired comparisons across multiple outcomes and time points without clear adjustment for multiple testing is reported. Please justify the use of paired t-tests for these repeated within-person measures, describe the assumed correlation structure, and address how multiplicity was handled to reduce the risk of false-positive findings.

Response: We thank the reviewer for this important comment. In the original submission, paired t-tests were used primarily to provide descriptive context for the trajectory plots. In response, we have substantially revised the analytic approach by incorporating formal longitudinal difference-in-differences (DiD) analyses using a two-way fixed-effects framework. These models account for the repeated-measures nature of the data by including individual fixed effects and by estimating standard errors clustered at both the matched-pair and individual levels, thereby accounting for within-person correlation across survey waves as well as dependence induced by the matched design. To address multiple testing, we applied the Benjamini-Hochberg false discovery rate (FDR) procedure across outcomes within each post-diagnosis period. Table 2 now reports estimates, 95% confidence intervals, and FDR-adjusted p-values for all DiD analyses.

We have clarified these procedures in the Methods section and revised the Results section to emphasize the model-based estimates. We believe these changes substantially reduce concerns regarding multiple testing while providing a more rigorous longitudinal assessment of differences between cancer survivors and matched controls over time.

3. There are several numerical inconsistencies in the Results that should be corrected for clarity. The text states that there were 5,431 incident cases, 7 with missing race/ethnicity, and 5,423 successfully matched among the remaining 5,424 cases. Please explicitly account for the one unmatched case and ensure the numbers are internally consistent throughout. In addition, the reported out-of-pocket spending difference differs slightly between the abstract ($2,383 [95% CI $1,705–$3,061]) and the Results section ($2,382 with no confidence interval reported). These values should be harmonized. Finally, Figure 3 appears to show a substantially higher peak in out-of-pocket spending at diagnosis than the reported between-group difference of approximately $2,382. Please clarify whether the reported estimate reflects an adjusted contrast while the figure displays raw values.

Response: We thank the reviewer for the careful review. We have revised the manuscript to explicitly stated that one case was not matched (first paragraph of Results): “One case could not be matched according to the prespecified matching criteria and was excluded from subsequent analyses.”

We also harmonized the reported out-of-pocket spending estimates throughout the manuscript. The abstract and Results section now consistently report the same estimate and corresponding 95% confidence interval.

Regarding Figure 3, the reported difference of $2,382 is correct ($5779 for the case group and $3397 for the control group). The figure may visually exaggerate the magnitude of the difference because the y-axis does not begin at zero. We have clarified this in the figure caption to avoid potential misinterpretation.

4. The interpretation of out-of-pocket spending would benefit from additional clarification. Because medical expenditures are reported over the prior two years, it is unclear how precisely spending can be aligned to the time of diagnosis, particularly when the primary finding appears to be a peak around t₀. In addition, decedent exit interviews may capture substantial end-of-life expenditures, which could disproportionately affect the cancer cohort and influence the observed spending gap. Please clarify how expenditures from exit interviews were incorporated into the spending trajectories and discuss the potential impact of the two-year recall window on temporal alignment of costs.

Response: We thank the reviewer for this important comment. We agree that it is important to consider the HRS expenditure reporting window for the out-of-pocket spending. In the HRS core interview, out-of-pocket medical expenditures represent expenditures incurred during the previous two years. Therefore, spending estimates assigned to a given survey wave should be interpreted as expenditures accumulated over the corresponding inter-survey period rather than costs occurring at a specific point in time. We have clarified this point by adding the reference period in the Methods section and using more precise language when describing out-of-pocket spending in the Results section.

The spending trajectories in our analysis were based exclusively on the out-of-pocket expenditure measure collected in the core HRS interviews. We did not use expenditure information obtained from exit interviews. We realized that the sentence, “At each follow-up, exit interviews were conducted with a family member or other informant for deceased participants to obtain relevant information (e.g., medical expenditures) during the final stages of life,” could have inadvertently implied that exit interview expenditure data were incorporated into our analysis. To avoid this confusion, we have removed this sentence from the revised manuscript.

Minor Comments

1. The discussion of self-reported cancer diagnosis should acknowledge that misclassification may be influenced by cognitive status, which is particularly relevant to the cognition findings.

Response: We agree with the reviewer that reliance on self-reported cancer diagnoses may introduce misclassification, and that such misclassification could be influenced by cognitive status. In the revised manuscript, we have explicitly acknowledged this limitation (section 4.3). We now note that “the misclassification may be differentially influenced by cognitive status, as individuals with cognitive impairment may be less likely to accurately report a prior diagnosis.” We appreciate this important suggestion, which has helped us provide a more nuanced interpretation of our findings.

2. Please correct "hermetic effects" to "hormetic effects."

Response: We have corrected the typo. Thank you!

3. The transient decline in BMI around diagnosis may reflect cancer-related weight loss rather than behavioral changes and warrants brief discussion.

Response: We thank the reviewer for raising this point. We have added “The transient decline in BMI observed around the time of diagnosis may reflect cancer-related weight loss or treatment effects rather than intentional behavioral changes.” In the revised manuscript (section 4.1).

4. References 26 and 60 do not appear to be appropriate primary scientific sources and should be replaced with peer-reviewed references or removed.

Response: We have removed these references.

5. Sensitivity analyses are referenced as available upon request. Consider including these results in the Supplementary Materials.

Response: We thank the reviewer for the suggestion. We have added results of sensitivity analyses in Supplementary Materials (Figure S1 and S2).

6. Please clarify the scoring of depressive symptoms, as the reported means are difficult to interpret from the current description.

Response: The score of depressive symptoms ranges from 0 to 8, with higher scores indicating greater numbers of symptoms. We have added this information in the revised manuscript (section 3). It is also presented in Table 1 along with survey questions.

7. The Abstract should more clearly describe the matching approach, including propensity score matching and the matching ratio.

Response: We have added this information to the Abstract. The relevant text now reads “Using 15 waves (1992–2022) of the Health and Retirement Study, we identified 5,423 incident cancer cases and matched them to up to four controls (n=17,530) using exact matching on sex and race/ethnicity and propensity score matching on age and entry wave (caliper=0.1).”

8. Consider reporting absolute percentage point changes alongside relative increases in functional limitations.

Response: We have added absolute percentage point changes to the text, which now reads “The proportion of participants experiencing functional limitations increased from 18% at t-1 to 26% at t0 (an 8-percentage-point [pp] increase; 44% relative increase) for basic activities and from 15% to 24% (a 9 pp increase; 60% relative increase) for instrumental activities.” (Section 3)

9. The reported timing of recovery in physical activity appears inconsistent (4 years vs. 6 years) and should be reconciled throughout the manuscript.

Response: We thank the reviewer for pointing out the inconsistency. We have revised the text, so it is consistently 6 years throughout the manuscript.

**Requests from Editors:**

1.Please ensure that your manuscript meets PLOS ONE's style requirements, including those for file naming. The PLOS ONE style templates can be found at

<https://journals.plos.org/plosone/s/file?id=wjVg/PLOSOne_formatting_sample_main_body.pdf> and

<https://journals.plos.org/plosone/s/file?id=ba62/PLOSOne_formatting_sample_title_authors_affiliations.pdf>

Response: Thank you for the templates and guidance. We have formatted the manuscript according to these requirements, including naming conventions.

2. Please note that funding information should not appear in any section or other areas of your manuscript. We will only publish funding information present in the Funding Statement section of the online submission form. Please remove any funding-related text from the manuscript.

Response: We have removed the Funding Statement from the main text.

3. Thank you for stating the following financial disclosure:

“The study is supported by Massey Comprehensive Cancer Center of Virginia Commonwealth University with funding from the National Cancer Institute (P30CA016059).”

We note that one or more of the authors is affiliated with the funding organization, indicating the funder may have had some role in the design, data collection, analysis or preparation of your manuscript for publication; in other words, the funder played an indirect role through the participation of the co-authors. If the funding organization did not play a role in the study design, data collection and analysis, decision to publish, or preparation of the manuscript and only provided financial support in the form of authors' salaries and/or research materials, please do the following:

1. Review your statements relating to the author contributions, and ensure you have specifically and accurately indicated the role(s) that these authors had in your study. These amendments should be made in the online form.

Response: We have reviewed the author contributions and confirm that they accurately reflect each author's role in the study.

2. Confirm in your cover letter that you agree with the following statement, and we will change the online submission form on your behalf:

“The funder provided support in the form of salaries for authors [insert relevant initials], but did not have any additional role in the study design, data collection and analysis, decision to publish, or preparation of the manuscript. The specific roles of these authors are articulated in the ‘author contributions’ section.

Response: We have added this statement in the cover letter.

4. Please include captions for your Supporting Information files at the end of your manuscript, and update any in-text citations to match accordingly. Please see our Supporting Information guidelines for more information: <http://journals.plos.org/plosone/s/supporting-information>.

Response: Thank you for the instruction. We have updated in-text citations to Supporting Information files and included captions.

5. If the reviewer comments include a recommendation to cite specific previously published works, please review and evaluate these publications to determine whether they are relevant and should be cited. There is no requirement to cite these works unless the editor has indicated otherwise.

Response: Thank you. Noted.
