# Supplementary material for: Long-term comparative trajectories of health, behavioral, and economic outcomes: From pre-diagnosis to survivorship
Source: PLoS One. 2026 Sep 9;21(9):e0357917. doi: 10.1371/journal.pone.0357917 (PMC13557411; doi:10.1371/journal.pone.0357917)
Supplement: Peer review history

# Peer review: Decision Letter 0

**DOI:** 10.1371/journal.pone.0357917.r001

**Authors:** Taiwo Opeyemi Aremu

17 Jun 2026

Dear Dr. Cheng,

Thank you for submitting your manuscript to PLOS ONE. After careful consideration, we feel that it has merit but does not fully meet PLOS ONE’s publication criteria as it currently stands. Therefore, we invite you to submit a revised version of the manuscript that addresses the points raised during the review process.

Please submit your revised manuscript by Aug 01 2026 11:59PM. If you will need more time than this to complete your revisions, please reply to this message or contact the journal office at plosone@plos.org. When you're ready to submit your revision, log on to https://www.editorialmanager.com/pone/ and select the 'Submissions Needing Revision' folder to locate your manuscript file.

- A letter that responds to each point raised by the academic editor and reviewer(s). You should upload this letter as a separate file labeled 'Response to Reviewers'.
- A marked-up copy of your manuscript that highlights changes made to the original version. You should upload this as a separate file labeled 'Revised Manuscript with Track Changes'.
- An unmarked version of your revised paper without tracked changes. You should upload this as a separate file labeled 'Manuscript'.

If you would like to make changes to your financial disclosure, please include your updated statement in your cover letter. Guidelines for resubmitting your figure files are available below the reviewer comments at the end of this letter.

If applicable, we recommend that you deposit your laboratory protocols in protocols.io to enhance the reproducibility of your results. Protocols.io assigns your protocol its own identifier (DOI) so that it can be cited independently in the future. For instructions see: https://journals.plos.org/plosone/s/submission-guidelines#loc-laboratory-protocols. Additionally, PLOS ONE offers an option for publishing peer-reviewed Lab Protocol articles, which describe protocols hosted on protocols.io. Read more information on sharing protocols at https://plos.org/protocols?utm_medium=editorial-email&utm_source=authorletters&utm_campaign=protocols.

As the corresponding author, your ORCID iD is verified in the submission system and will appear in the published article. PLOS supports the use of ORCID, and we encourage all coauthors to register for an ORCID iD and use it as well. Please encourage your coauthors to verify their ORCID iD within the submission system before final acceptance, as unverified ORCID iDs will not appear in the published article. *Only*  the individual author can complete the verification step; PLOS staff *cannot*  verify ORCID iDs on behalf of authors.

We look forward to receiving your revised manuscript.

Kind regards,

Taiwo Opeyemi Aremu, MD, MPH, PhD

Academic Editor

PLOS One

Journal Requirements:

When submitting your revision, we need you to address these additional requirements.

1.Please ensure that your manuscript meets PLOS ONE's style requirements, including those for file naming. The PLOS ONE style templates can be found at

https://journals.plos.org/plosone/s/file?id=wjVg/PLOSOne_formatting_sample_main_body.pdf and

https://journals.plos.org/plosone/s/file?id=ba62/PLOSOne_formatting_sample_title_authors_affiliations.pdf

2. Please note that funding information should not appear in any section or other areas of your manuscript. We will only publish funding information present in the Funding Statement section of the online submission form. Please remove any funding-related text from the manuscript.

3. Thank you for stating the following financial disclosure:

“The study is supported by Massey Comprehensive Cancer Center of Virginia Commonwealth University with funding from the National Cancer Institute (P30CA016059).”

We note that one or more of the authors is affiliated with the funding organization, indicating the funder may have had some role in the design, data collection, analysis or preparation of your manuscript for publication; in other words, the funder played an indirect role through the participation of the co-authors. If the funding organization did not play a role in the study design, data collection and analysis, decision to publish, or preparation of the manuscript and only provided financial support in the form of authors' salaries and/or research materials, please do the following:

1. Review your statements relating to the author contributions, and ensure you have specifically and accurately indicated the role(s) that these authors had in your study. These amendments should be made in the online form.

2. Confirm in your cover letter that you agree with the following statement, and we will change the online submission form on your behalf:

“The funder provided support in the form of salaries for authors [insert relevant initials], but did not have any additional role in the study design, data collection and analysis, decision to publish, or preparation of the manuscript. The specific roles of these authors are articulated in the ‘author contributions’ section.

4. Please include captions for your Supporting Information files at the end of your manuscript, and update any in-text citations to match accordingly. Please see our Supporting Information guidelines for more information: http://journals.plos.org/plosone/s/supporting-information.

5. If the reviewer comments include a recommendation to cite specific previously published works, please review and evaluate these publications to determine whether they are relevant and should be cited. There is no requirement to cite these works unless the editor has indicated otherwise.

[Note: HTML markup is below. Please do not edit.]

Reviewers' comments:

Reviewer's Responses to Questions

**Comments to the Author**

1. Is the manuscript technically sound, and do the data support the conclusions?

Reviewer #1: Partly

Reviewer #2: Yes

**********

2. Has the statistical analysis been performed appropriately and rigorously?

Reviewer #1: No

Reviewer #2: Yes

**********

3. Have the authors made all data underlying the findings in their manuscript fully available?

The PLOS Data policy

Reviewer #1: Yes

Reviewer #2: Yes

**********

4. Is the manuscript presented in an intelligible fashion and written in standard English?

Reviewer #1: Yes

Reviewer #2: Yes

**********

Reviewer #1: This manuscript addresses an important and timely topic in cancer survivorship, examining the long-term trajectories of health, behavioural and economic outcomes using a large nationally representative longitudinal dataset (the Health and Retirement Study, HRS). The study design, which is based on a matched cohort approach and an extended follow-up period, is a clear strength that provides valuable insights into multidimensional survivorship patterns over time. The manuscript is well structured and written in standard academic English, and the topic is clearly relevant to public health and policy.

However, there are several important methodological and analytical issues that need to be addressed. Firstly, the statistical analysis is largely descriptive and does not make full use of the longitudinal nature of the data. The absence of advanced modelling approaches, such as mixed-effects models or Generalised Estimating Equations (GEE), limits the robustness and interpretability of the findings. Secondly, although the matching strategy is appropriate, it does not account for key potential confounders such as comorbidities, socioeconomic status or baseline health conditions. This raises concerns about residual confounding. Thirdly, the observational design precludes causal inference; however, some of the interpretations in the manuscript appear to use causal language and should therefore be revised.

Additionally, key sources of bias, such as survivorship, attrition, and measurement biases related to self-reported data, are not adequately addressed in the limitations section. The lack of clinical granularity, particularly with regard to cancer type, stage and treatment modalities, further limits the applicability and interpretability of the findings. The results section would benefit from more detailed statistical reporting, including effect sizes and multivariable analyses. While the discussion is comprehensive, it includes some speculative explanations that are not directly supported by the data and should be tempered.

In terms of transparency, the data availability statement is appropriate given that the HRS dataset is publicly accessible. However, providing additional details about dataset versions and analytical procedures would strengthen reproducibility further. While the reference list is generally relevant and up to date, improvements in formatting consistency and the removal of non-academic sources are recommended.

No major concerns relating to research ethics, dual publication or publication ethics were identified. Overall, the manuscript shows strong potential, but substantial revisions are required to improve its methodological rigour, analytical depth and clarity of interpretation before it can be considered for publication.

Reviewer #2: This study uses a large, well-established longitudinal dataset to examine health trajectories before and after cancer diagnosis across multiple domains. The long follow-up period, matched-cohort design, and broad range of outcomes are notable strengths. However, several methodological and interpretive concerns should be addressed before the manuscript is suitable for publication.

Major Concerns

1. The reported finding of better cognitive function among cancer survivors may be influenced by survivor bias. Patients with cancer who have poorer cognition may be more likely to die and leave the analysis, resulting in a progressively healthier surviving cohort over time. The comparison of cognition–mortality associations between groups does not fully address this concern. Please discuss the potential impact of differential survival on the cognitive findings and consider additional analyses (e.g. sensitivity analyses) to assess the robustness of this result. At minimum, the cognitive findings should be interpreted more cautiously and framed as hypothesis-generating.

2. A large number of repeated paired comparisons across multiple outcomes and time points without clear adjustment for multiple testing is reported. Please justify the use of paired t-tests for these repeated within-person measures, describe the assumed correlation structure, and address how multiplicity was handled to reduce the risk of false-positive findings.

3. There are several numerical inconsistencies in the Results that should be corrected for clarity. The text states that there were 5,431 incident cases, 7 with missing race/ethnicity, and 5,423 successfully matched among the remaining 5,424 cases. Please explicitly account for the one unmatched case and ensure the numbers are internally consistent throughout. In addition, the reported out-of-pocket spending difference differs slightly between the abstract ($2,383 [95% CI $1,705–$3,061]) and the Results section ($2,382 with no confidence interval reported). These values should be harmonized. Finally, Figure 3 appears to show a substantially higher peak in out-of-pocket spending at diagnosis than the reported between-group difference of approximately $2,382. Please clarify whether the reported estimate reflects an adjusted contrast while the figure displays raw values.

4. The interpretation of out-of-pocket spending would benefit from additional clarification. Because medical expenditures are reported over the prior two years, it is unclear how precisely spending can be aligned to the time of diagnosis, particularly when the primary finding appears to be a peak around t₀. In addition, decedent exit interviews may capture substantial end-of-life expenditures, which could disproportionately affect the cancer cohort and influence the observed spending gap. Please clarify how expenditures from exit interviews were incorporated into the spending trajectories and discuss the potential impact of the two-year recall window on temporal alignment of costs.

Minor Comments

1. The discussion of self-reported cancer diagnosis should acknowledge that misclassification may be influenced by cognitive status, which is particularly relevant to the cognition findings.

2. Please correct "hermetic effects" to "hormetic effects."

3. The transient decline in BMI around diagnosis may reflect cancer-related weight loss rather than behavioral changes and warrants brief discussion.

4. References 26 and 60 do not appear to be appropriate primary scientific sources and should be replaced with peer-reviewed references or removed.

5. Sensitivity analyses are referenced as available upon request. Consider including these results in the Supplementary Materials.

6. Please clarify the scoring of depressive symptoms, as the reported means are difficult to interpret from the current description.

7. The Abstract should more clearly describe the matching approach, including propensity score matching and the matching ratio.

8. Consider reporting absolute percentage point changes alongside relative increases in functional limitations.

9. The reported timing of recovery in physical activity appears inconsistent (4 years vs. 6 years) and should be reconciled throughout the manuscript.

**********

what does this mean?). If published, this will include your full peer review and any attached files.

If you choose “no”, your identity will remain anonymous but your review may still be made public.

**Do you want your identity to be public for this peer review?**  For information about this choice, including consent withdrawal, please see our Privacy Policy

Reviewer #1: No

Reviewer #2: No

**********

[NOTE: If reviewer comments were submitted as an attachment file, they will be attached to this email and accessible via the submission site. Please log into your account, locate the manuscript record, and check for the action link "View Attachments". If this link does not appear, there are no attachment files.]

To ensure your figures meet our technical requirements, please review our figure guidelines: https://journals.plos.org/plosone/s/figures

You may also use PLOS’s free figure tool, NAAS, to help you prepare publication quality figures: https://journals.plos.org/plosone/s/figures#loc-tools-for-figure-preparation.

NAAS will assess whether your figures meet our technical requirements by comparing each figure against our figure specifications.

Attachment Submitted filename: review report.docx

# Author comment: Author response to Decision Letter 1

**DOI:** 10.1371/journal.pone.0357917.r002

27 Jul 2026

Please see our uploaded file for responses to comments.

Attachment Submitted filename: Response to comments R1.docx

# Peer review: Decision Letter 1

**DOI:** 10.1371/journal.pone.0357917.r003

**Authors:** Taiwo Opeyemi Aremu

24 Aug 2026

Long-Term Comparative Trajectories of Health, Behavioral, and Economic Outcomes: From Pre-Diagnosis to Survivorship

PONE-D-25-60630R1

Dear Dr. Cheng,

We’re pleased to inform you that your manuscript has been judged scientifically suitable for publication and will be formally accepted for publication once it meets all outstanding technical requirements.

Within one week, you’ll receive an e-mail detailing the required amendments. When these have been addressed, you’ll receive a formal acceptance letter and your manuscript will be scheduled for publication.

An invoice will be generated when your article is formally accepted. Please note, if your institution has a publishing partnership with PLOS and your article meets the relevant criteria, all or part of your publication costs will be covered. Please make sure your user information is up-to-date by logging into Editorial Manager at Editorial Manager® and clicking the ‘Update My Information' link at the top of the page. For questions related to billing, please contact billing support.

If your institution or institutions have a press office, please notify them about your upcoming paper to help maximize its impact. If they’ll be preparing press materials, please inform our press team as soon as possible -- no later than 48 hours after receiving the formal acceptance. Your manuscript will remain under strict press embargo until 2 pm Eastern Time on the date of publication. For more information, please contact onepress@plos.org.

Kind regards,

Taiwo Opeyemi Aremu, MD, MPH, PhD

Academic Editor

PLOS One

Additional Editor Comments (optional):

Reviewers' comments:

Reviewer's Responses to Questions

**Comments to the Author**

Reviewer #2: All comments have been addressed

**********

2. Is the manuscript technically sound, and do the data support the conclusions?

Reviewer #2: Yes

**********

3. Has the statistical analysis been performed appropriately and rigorously?

Reviewer #2: Yes

**********

4. Have the authors made all data underlying the findings in their manuscript fully available?

The PLOS Data policy

Reviewer #2: Yes

**********

5. Is the manuscript presented in an intelligible fashion and written in standard English?

Reviewer #2: Yes

**********

Reviewer #2: (No Response)

**********

what does this mean?). If published, this will include your full peer review and any attached files.

If you choose “no”, your identity will remain anonymous but your review may still be made public.

**Do you want your identity to be public for this peer review?**  For information about this choice, including consent withdrawal, please see our Privacy Policy

Reviewer #2: No

**********

# Editor report: Acceptance letter

**DOI:** 10.1371/journal.pone.0357917.r004

**Authors:** Taiwo Opeyemi Aremu

PONE-D-25-60630R1

PLOS One

Dear Dr. Cheng,

I'm pleased to inform you that your manuscript has been deemed suitable for publication in PLOS One. Congratulations! Your manuscript is now being handed over to our production team.

At this stage, our production department will prepare your paper for publication. This includes ensuring the following:

* All references, tables, and figures are properly cited

* All relevant supporting information is included in the manuscript submission,

* There are no issues that prevent the paper from being properly typeset

You will receive further instructions from the production team, including instructions on how to review your proof when it is ready. Please keep in mind that we are working through a large volume of accepted articles, so please give us a few days to review your paper and let you know the next and final steps.

Lastly, if your institution or institutions have a press office, please let them know about your upcoming paper now to help maximize its impact. If they'll be preparing press materials, please inform our press team within the next 48 hours. Your manuscript will remain under strict press embargo until 2 pm Eastern Time on the date of publication. For more information, please contact onepress@plos.org.

You will receive an invoice from PLOS for your publication fee after your manuscript has reached the completed accept phase. If you receive an email requesting payment before acceptance or for any other service, this may be a phishing scheme. Learn how to identify phishing emails and protect your accounts at https://explore.plos.org/phishing.

If we can help with anything else, please email us at customercare@plos.org.

Thank you for submitting your work to PLOS One and supporting open access.

Kind regards,

PLOS One Editorial Office Staff

on behalf of

Dr. Taiwo Opeyemi Aremu

Academic Editor

PLOS One
